# Penicillin-Binding Protein 1 (PBP1) of Staphylococcus aureus Has Multiple Essential Functions in Cell Division

**DOI:** 10.1128/mbio.00669-22

**Published:** 2022-06-15

**Authors:** Katarzyna Wacnik, Vincenzo A. Rao, Xinyue Chen, Lucia Lafage, Manuel Pazos, Simon Booth, Waldemar Vollmer, Jamie K. Hobbs, Richard J. Lewis, Simon J. Foster

**Affiliations:** a School of Biosciences, University of Sheffield, Sheffield, United Kingdom; b The Florey Institute for Host-Pathogen Interactions, University of Sheffield, Sheffield, United Kingdom; c Biosciences Institute, Newcastle University, Newcastle upon Tyne, United Kingdom; d Department of Physics and Astronomy, University of Sheffield, Sheffield, United Kingdom; e Centre for Bacterial Cell Biology, Bioscience Institute, Newcastle University, Newcastle upon Tyne, United Kingdom; McMaster University

**Keywords:** *Staphylococcus aureus*, cell division, penicillin-binding proteins, peptidoglycan

## Abstract

Bacterial cell division is a complex process requiring the coordination of multiple components to allow the appropriate spatial and temporal control of septum formation and cell scission. Peptidoglycan (PG) is the major structural component of the septum, and our recent studies in the human pathogen Staphylococcus aureus have revealed a complex, multistage PG architecture that develops during septation. Penicillin-binding proteins (PBPs) are essential for the final steps of PG biosynthesis; their transpeptidase activity links the peptide side chains of nascent glycan strands. PBP1 is required for cell division in S. aureus, and here, we demonstrate that it has multiple essential functions associated with its enzymatic activity and as a regulator of division. Loss of PBP1, or just its C-terminal PASTA domains, results in cessation of division at the point of septal plate formation. The PASTA domains can bind PG and thereby potentially coordinate the cell division process. The transpeptidase activity of PBP1 is also essential, but its loss leads to a strikingly different phenotype of thickened and aberrant septa, which is phenocopied by the morphological effects of adding the PBP1-specific β-lactam, meropenem. Together, these results lead to a model for septal PG synthesis where PBP1 enzyme activity is required for the characteristic architecture of the septum and PBP1 protein molecules enable the formation of the septal plate.

## INTRODUCTION

Peptidoglycan (PG) is the major structural component of the bacterial cell wall and is essential for maintaining cell shape, integrity, and survival (1–3). The final stages of assembly of this large polymeric molecule are mediated by penicillin-binding proteins (PBPs), key PG synthases that, through their transglycosylase (TG) and transpeptidase (TP) activities, polymerize glycan chains and cross-link them into a mesh-like hydrogel (4, 5). Since the cell wall is essential for maintaining bacterial life, PBPs and PG synthesis are a target of some of the most important antibiotics, β-lactams (penicillins) and glycopeptides (vancomycin) (6, 7). The major human pathogen Staphylococcus aureus has a minimalist PBP system, as it encodes only four PBPs, PBP1 to PBP4 (8). Only PBP1 (class B PBP with only TP activity, bPBP) and PBP2 (class A bifunctional PBP with both TG and TP activities, aPBP) are essential and sufficient for septal and peripheral PG synthesis in S. aureus (8, 9). PBP2 is the major PG synthase of S. aureus, and the septum formation activity of PBP2 is mediated by its substrate, lipid II (10). Although PBP2 is essential, loss of its TP activity can be compensated for by a horizontally acquired class B PBP2A in methicillin-resistant S. aureus (MRSA) (11). PBP2A, however, cannot replace PBP1, whose loss is detrimental to the viability of S. aureus (12). PBP1 and PBP3 (bPBP) form cognate pairs with the monofunctional TGs, FtsW and RodA, belonging to the SEDS (shape, elongation, division, and sporulation) family (13) to facilitate septum formation (PBP1-FtsW) and to maintain the prolate cell shape (PBP3-RodA) of S. aureus, respectively (14). Activation of the transglycosylase activity of FtsW requires complex formation with PBP1 (15). PBP4 is a class C PBP with d,d-carboxypeptidase activity (cPBP) and has a TP activity that contributes to the high-level cross-linking of PG and MRSA resistance to β-lactams (16, 17).

The cell wall of Gram-positive bacteria is decorated with wall teichoic acid (WTA) glycopolymers (18). WTA regulates cell shape, ion homeostasis, autolytic enzymes, growth, and division (19). In S. aureus, WTA plays a crucial role in virulence, MRSA resistance to β-lactam antibiotics, PBP4 localization at the septum, and PG cross-linking (20–23).

Although S. aureus PBPs have been studied over many years, the specific roles of PBP1 in cell division, PG synthesis, and architecture are not well understood. Previous studies have shown that while PBP1 is essential, its TP activity is not, implying another role (12, 14). However, this work was performed in an MRSA background that contains PBP2A, encoded by *mecA*, which is non-native to S. aureus (24). While PBP2A cannot replace PBP1, how these proteins interact is unknown. We have recently shown that the presence of *mecA* has a profound effect on cellular physiology (25). Thus, it is important to understand individual and combined roles of S. aureus PBPs in both the presence and absence of the exogenous PBP2A, as the vast majority of S. aureus infections are caused by methicillin-sensitive strains.

## RESULTS

### S. aureus PBP1 PASTA domains are essential for growth and PBP1 functionality.

**FIG 1:**
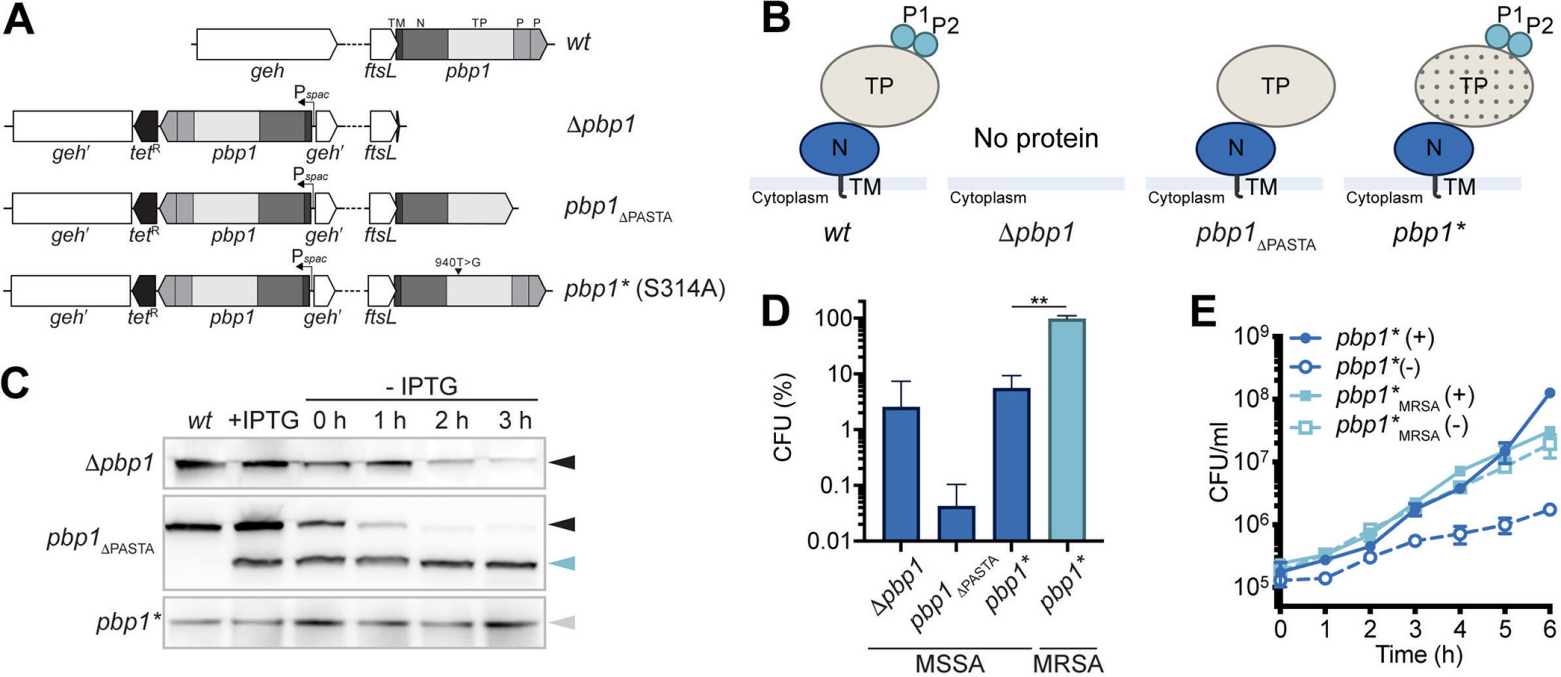
PBP1, its PASTA domains, and transpeptidase activity are essential in MSSA. (A) Schematic representation of genetic constructs used in this study. In S. aureus WT (*wt*) the 5′ end of *pbp1* overlaps with the 3′ end of *ftsL*. The *pbp1* gene encodes a protein containing the short cytoplasmic tail, transmembrane helix (TM), N-terminal pedestal domain (N), transpeptidase (TP) domain, and two PASTA domains (P1 and P2). In the mutants, an ectopic copy of *pbp1* is placed under the control of the P*spac* promoter at the lipase (*geh*) locus, whereas the gene in the native *pbp1* locus is either deleted (Δ*pbp1*), has P1 and P2 domains removed (*pbp1*_ΔPASTA_), or has a point mutation which results in inactivation of the TP domain (pbp1*). (B) Schematic representation of the domain architecture of PBP1 in S. aureus WT (*wt*) and PBP1 forms produced by Δ*pbp1*, *pbp1*_ΔPASTA_, and pbp1* mutants in the absence of inducer. The TP domain inactivation is shown by dotted shading. (C) Immunoblot showing PBP1 levels in SH1000 *lacI* (*wt*) and in Δ*pbp1*, *pbp1*_ΔPASTA_, and pbp1* grown with IPTG (+IPTG) and for 0, 1, 2, and 3 h without inducer (–IPTG) analyzed using anti-PBP1 antibody. Expected sizes: PBP1 and PBP1* = 83 kDa (black and gray arrowheads, respectively) and PBP1_ΔPASTA_ = 67 kDa (light blue arrowhead). (D) Plating efficiency of Δ*pbp1*, *pbp1*_ΔPASTA_, and pbp1* (MSSA) cells and MRSA pbp1* (pbp1*_MRSA_) cells upon inducer removal compared to the control groups grown in the presence of inducer. The *P* value was determined by Mann-Whitney *U* tests. *P = *0.0043 (**, *P < *0.01). Data represent the mean ± standard deviation (SD). (E) Growth curves of pbp1* (MSSA) and MRSA pbp1*(pbp1*_MRSA_) in the presence (+) or absence (–) of IPTG. Data represent the mean ± SD. Error bars that are smaller than the symbols are not shown. Data are representative of three (C and E) and at least four (D) independent experiments.

PBP1 has a short cytoplasmic fragment, a membrane-spanning sequence, an exocytoplasmic N-terminal pedestal domain, and a C-terminal region consisting of the TP domain and two PASTA domains (for penicillin-binding protein and serine/threonine kinase-associated domain) (26, 27). We created a set of conditional mutants of *pbp1* to investigate the role of PBP1 in cell division and PG synthesis. An ectopic copy of *pbp1* under the control of the P*spac* promoter (P*_spac_*-*pbp1*) was placed at the lipase locus (*geh*::P*_spac_*-*pbp1*) of S. aureus SH1000, and a series of changes were made in this genetic background at the native *pbp1* locus: (i) an in-frame deletion of *pbp1* (Δ*pbp1*), (ii) a deletion of the region encoding the two PASTA domains (*pbp1*_ΔPASTA_), and (iii) the substitution of the catalytic Ser314 to Ala in the TP domain (pbp1*) (Fig. 1A and B). We examined the essentiality of PBP1, the PASTA domains, and the active TP domain with these mutants. Depletion of PBP1 via IPTG (isopropyl-β-d-thiogalactopyranoside) removal (Fig. 1C and Fig. S1A and B) resulted in cell death, confirming the essentiality of PBP1 (Fig. 1C and D and Fig. S1C and D). Deletion of the PASTA domains also led to growth inhibition and more than 99% cell death within 4 h (Fig. 1D and Fig. S1C and D). Importantly, this phenotype was not associated with PBP1_ΔPASTA_ instability (Fig. 1C and Fig. S1A) or loss of its ability to bind its substrate analogue BocillinFL (Fig. S1B). In contrast, deletion of the PASTA domains of Streptococcus pneumoniae PBP2x, a PBP1 orthologue, resulted in a complete loss of BocillinFL binding (28). These results indicate that the PASTA domains are essential for S. aureus growth and PBP1 functionality but not its stability.

10.1128/mbio.00669-22.1 FIG S1 Essentiality of PBP1, PASTA, and TP domains in S. aureus. (A) Relative levels of PBP1 in Δ*pbp1*, *pbp1*_ΔPASTA_, and pbp1* grown with IPTG and for 0, 1, 2, and 3 h after inducer removal. PBP1 levels were normalized to protein levels in SH1000 *lacI* (*wt*). PBP1_ΔPASTA_ levels were normalized to PBP1_ΔPASTA_ levels in the presence of inducer. Quantifications are for the data shown in Fig. 1C. Data represent the mean ± SD. (B) BocillinFL gel-based analysis of penicillin-binding proteins in SH1000 *lacI* (*wt*) and Δ*pbp1*, *pbp1*_ΔPASTA_, and pbp1* grown with IPTG and 0, 1, 2, and 3 h after inducer removal. (C) Growth of Δ*pbp1*, *pbp1*_ΔPASTA_, and pbp1* with or without IPTG. Quantifications for the plate assay are shown in Fig. 1D. (D) Growth curves of Δ*pbp1*, *pbp1*_ΔPASTA_, and pbp1* in the presence or absence of IPTG. Data represent the mean ± SD. Error bars that are smaller than the data point symbols are not shown. (E) Schematic representation of the *pbp1*_STOP_ mutant. A SNP (874 G for T) resulted in a premature stop codon (E292X) and removal of the TP and PASTA domains. (F) Schematic representation of the domain architecture of PBP1_STOP_ encoded by the *pbp1*_STOP_ mutant. (G) Immunoblot showing PBP1 levels in SH1000 *lacI* (*wt*) and *pbp1*_STOP_ grown with IPTG and for 0, 1, 2, and 3 h without inducer analyzed using anti-PBP1 antibody. Expected sizes: PBP1 = 83 kDa (black arrowhead) and PBP1_STOP_ = 33 kDa (red arrowhead). Data are representative of two (B and G), three (A and D), and at least four (C) independent experiments. Download FIG S1, TIF file, 1.6 MB. Copyright © 2022 Wacnik et al. 2022 Wacnik et al. https://creativecommons.org/licenses/by/4.0/ This content is distributed under the terms of the Creative Commons Attribution 4.0 International license.

During construction of the pbp1* mutant we obtained, by serendipity, a *pbp1*_STOP_ mutant in which a single-nucleotide polymorphism (SNP) in the codon for Glu292 resulted in its replacement with a premature stop codon and the truncation of the entire TP and PASTAs region of PBP1 (Fig. S1E and F). However, immunoblot analysis using anti-PBP1 sera could not confirm the presence of the PBP1_STOP_ protein in the *pbp1*_STOP_ mutant (Fig. S1G), suggesting that stability of the N-terminal domain of PBP1 is dependent on its C terminus but not the PASTA domains. Although inactivation of PBP1 TP activity (PBP1*) did not affect protein stability (Fig. 1C), it did remove the ability of PBP1 to bind BocillinFL (Fig. S1B). The loss of PBP1 TP activity resulted in severely compromised growth on solid medium (Fig. 1D and Fig. S1C) and reduced cellular viability in liquid culture (Fig. 1E and Fig. S1D). Thus, the TP activity of PBP1 is required for growth in the SH1000 background. Inactivation of the PBP1 TP activity was previously reported not to affect growth in the COL strain background (14). The differences in the necessity for the PBP1 TP activity could result from COL being MRSA, whereas SH1000 is a methicillin-sensitive S. aureus (MSSA) strain.

### PBP1 TP activity is crucial in MSSA but not in MRSA.

We have recently developed a set of defined strains where high-level β-lactam resistance of MRSA is mediated by *mecA* encoding PBP2A and a mutation in either *rpoB* or *rpoC* (25). This combination of genetic alterations (*mecA^+^ rpoB*) is present in COL (25). To test if the apparent disparity in PBP1’s role is associated with MRSA, we developed a high-level resistant mutant of pbp1* in the well-characterized S. aureus SH1000 by adding the *mecA rpoB*^H929Q^ to the MSSA pbp1* mutant, resulting in SH1000_MRSA_ pbp1* (Fig. S2A). Inactivating PBP1 TP did not affect the ability of SH1000_MRSA_ pbp1* to grow in the absence of IPTG, whereas *pbp1* depletion led to growth inhibition in the isogenic Δ*pbp1* MSSA and MRSA strains (Fig. 1D and E and Fig. S2B to D). Thus, the fundamental role of PBP1 in growth and division can only be studied in an MSSA background, as otherwise, the role of PBP1 can be confounded by the presence of the MRSA resistance apparatus.

10.1128/mbio.00669-22.2 FIG S2 Essentiality of PBP1 and its TP activity in methicillin-resistant S. aureus. (A) Schematic representation of evolution of high-level β-lactam-resistant Δ*pbp1* and pbp1*. A single-copy *mecA* under its native promoter (p*mecA*) was introduced at the *lysA* locus, resulting in low-level oxacillin-resistant Δ*pbp1* p*mecA* and pbp1* p*mecA.* Subsequently, addition of a point mutation in the *rpoB* gene results in development of high-level oxacillin-resistant Δ*pbp1*_MRSA_ and pbp1*_MRSA_. Oxacillin MICs shown in brackets were measured using Etest strips. (B) Plating efficiency of Δ*pbp1* (MSSA) and MRSA Δ*pbp1* (Δ*pbp1*_MRSA_) cells upon inducer removal compared to the control groups grown in the presence of inducer. Data represent the mean ± SD. The *P* value was determined by Mann-Whitney *U* tests. *P = *0.5429 (NS, not significant). (C) Growth curves of Δ*pbp1*_MRSA_ and pbp1*_MRSA_ in the presence or absence of IPTG. Data represent the mean ± SD. Error bars that are smaller than the data point symbols are not shown. (D) Growth of Δ*pbp1*_MRSA_ and pbp1*_MRSA_ with or without IPTG. Quantifications for Δ*pbp1*_MRSA_ are shown in panel C and for pbp1*_MRSA_ are shown in Fig. 1B. Data are representative of at least three independent experiments. Download FIG S2, TIF file, 1.3 MB. Copyright © 2022 Wacnik et al. 2022 Wacnik et al. https://creativecommons.org/licenses/by/4.0/ This content is distributed under the terms of the Creative Commons Attribution 4.0 International license.

### PBP1 PASTA domains are required for septum progression.

**FIG 2:**
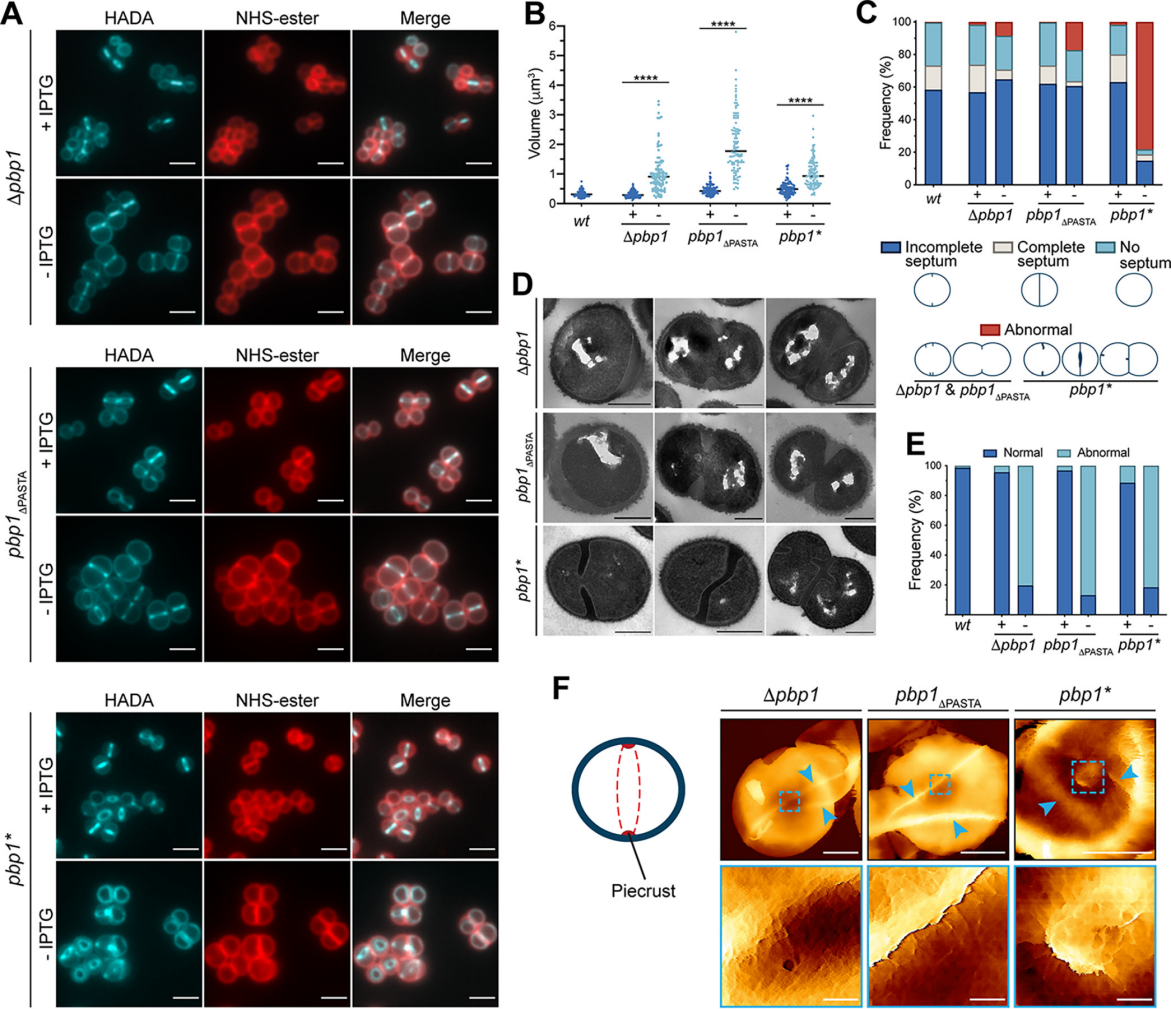
Role of PBP1 in cell division and PG synthesis in S. aureus. (A) Δ*pbp1*, *pbp1*_ΔPASTA_, and pbp1* grown with or without IPTG for 2 h, incubated with HADA for 5 min to show nascent PG, and counterlabeled with NHS-ester Alexa Fluor 555 to image the cell wall. Images are average intensity projections of z stacks. Scale bars = 2 μm. (B) Cell volumes of SH1000 *lacI* (*wt*) and Δ*pbp1*, *pbp1*_ΔPASTA_, and pbp1* grown with (+) or without (–) IPTG for 2 h as measured by fluorescence microscopy after NHS-ester Alexa Fluor 555 labeling. Each dot represents a single cell. The median of each distribution is indicated by a black line. The *P* value was determined by Mann-Whitney *U* tests (****, *P < *0.0001). From left to right, *P = *3.033e-033, 4.670e-049, and 2.206e-022. The number of cells analyzed for each mutant and condition was *n *≥ 100. (C) Quantification of cellular phenotypes for SH1000 *lacI* (*wt*) and Δ*pbp1*, *pbp1*_ΔPASTA_, and pbp1* based on HADA incorporation (panel A) after incubation with (+) or without (–) IPTG for 2 h. From left to right, *n *= 370, 427, 332, 314, 364, 512, and 331. (D) TEM of Δ*pbp1*, *pbp1*_ΔPASTA_, and pbp1* grown for 2 h in the absence of inducer. Scale bars = 500 nm. (E) Quantification of cellular phenotypes based on TEM data of SH1000 *lacI* (*wt*) and Δ*pbp1*, *pbp1*_ΔPASTA_, and pbp1* grown for 2 h in the presence (+) or absence (–) of IPTG. Examples of cells classified as normal (blue) are shown in Fig. S4B. Cells with abnormal phenotypes (light blue) are shown in panel D and Fig. S4C. From left to right, *n *= 304, 391, 329, 314, 377, 263, and 302. (F) AFM topographic images of internal surface of purified sacculi from Δ*pbp1*, *pbp1*_ΔPASTA_, and pbp1* grown in the absence of inducer for 2 h. (Left) Diagram of the inside of the cell before septal plate formation and (right) AFM images of sacculi (top images, scale bars = 500 nm, data scales [z]: 450, 300, and 100 nm from left to right, respectively) and higher magnification images (bottom images, scale bars = 50 nm, data scales [z]: 70, 100, and 50 nm from left to right, respectively) scanned within the boxed areas from the top images. The arrowheads indicate abnormal piecrusts. The boxed areas show details of piecrust features in Δ*pbp1* and *pbp1*_ΔPASTA_ and material agglomeration in pbp1*. Data are representative of two (D to F) and (A to C) three independent experiments.

PG synthesis still occurred in Δ*pbp1*, *pbp1*_ΔPASTA_, and pbp1* in the absence of IPTG, despite cell growth inhibition, as measured by the incorporation of the fluorescent d-amino acid derivative HADA (Fig. 2A). This was not a consequence of the nonsynthesis, exchange reaction carried out by PBP4, as it occurred in *pbp4* as well as with the dipeptide ADA-DA (9, 29) (Fig. S3). All variants increased in cell volume upon depletion of *pbp1*, whereas *pbp1*_ΔPASTA_ was enlarged by almost twice as much as Δ*pbp1* and pbp1* (Fig. 2A and B and Fig. S4A). Despite differences in cell size, both Δ*pbp1* and *pbp1*_ΔPASTA_ demonstrated a decrease in the proportion of cells with complete septa compared to the parent (Fig. 2A and C). Transmission electron microscopy (TEM) showed that more than 80% of the population had morphological defects, including cell wall thickening, PG blebs, and misshapen and/or multiple incomplete septa. (Fig. 2D and E and Fig. S4B and C). Such septa had abnormally thick bases and sharply pointed leading edges, suggesting that there is a problem with septal progression after initiation. Atomic force microscopy (AFM) previously revealed that the first step in cell division is the formation of a thick band of PG called the “piecrust” (30). Within this, the septal plate is formed, which has two PG architectures: disordered mesh facing the cell membrane and concentric rings in the septum core (5). Here, lack of PBP1 or the PBP1 PASTA domains led to formation of more than one, and often misplaced, piecrust. These mutations also caused an increase in unfinished septal annuli and alterations in the PG ring architecture (Fig. 2F and Fig. S5A to C, arrowheads), a feature that is revealed immediately after cell scission (5). Thus, depletion of PBP1 did not stop septum initiation, but the loss of the PASTA domains was enough to cause formation of irregular piecrusts, arrest septal plate formation, and lead to an altered septal PG architecture.

10.1128/mbio.00669-22.3 FIG S3 Loss of PBP1, PASTAs, or TP activity of PBP1 does not prevent PG synthesis. (A to C) PG incorporation in *pbp4* mutants depleted of PBP1. Δ*pbp1 pbp4*, *pbp1*_ΔPASTA_ *pbp4*, and pbp1* pbp4 grown with or without IPTG for 2 h and incubated with HADA for 5 min to show nascent PG incorporation. (D to F) Δ*pbp1*, *pbp1*_ΔPASTA_, and pbp1* grown with or without IPTG for 2 h, incubated with dipeptide (ADA-DA) for 5 min, and clicked to Atto 488 alkyne Alexa Fluor 488 to show nascent PG incorporation. Fluorescence images are average intensity projections of z stacks. Scale bars = 2 μm. Images are representatives of two independent experiments. Download FIG S3, TIF file, 2.5 MB. Copyright © 2022 Wacnik et al. 2022 Wacnik et al. https://creativecommons.org/licenses/by/4.0/ This content is distributed under the terms of the Creative Commons Attribution 4.0 International license.

10.1128/mbio.00669-22.4 FIG S4 Role of PBP1 in S. aureus. (A) Fluorescence images of WT (SH1000 *lacI*) labeled with HADA for 5 min (nascent PG) and counterlabeled with NHS-ester Alexa Fluor 555 (cell wall). Images are average intensity projections of z stacks. Scale bars = 2 μm. (B) TEM of WT (SH1000 *lacI*), Δ*pbp1*, *pbp1*_ΔPASTA_, and pbp1* grown in the presence of IPTG (where appropriate) categorized as normal phenotype (blue). Scale bars = 500 nm. (C) TEM of Δ*pbp1*, *pbp1*_ΔPASTA_, and pbp1* grown in the absence of IPTG for 2 h categorized as abnormal phenotype (1, PG blebs; 2, thickened cell wall; 3, thickened complete septum; 4, multiple septa; 5, misshapen incomplete septum; 6, thick incomplete septum with rounded leading edge; 7, curved septum; 8, separation defect). Scale bars = 500 nm. (D) Fluorescence images of pbp1* pbp3 pbp4 grown with or without IPTG for 2 h, labeled with HADA for 5 min (nascent PG), and counterstained with NHS-ester Alexa Fluor 555 (cell wall). Images are average intensity projections of z stacks. Scale bars = 2 μm. (E) Plating efficiency of pbp1* pbp3 pbp4 cells upon inducer removal compared to the control grown in the presence of inducer. The *P* value was determined by Mann-Whitney *U* tests. *P = *0.2619 (NS, not significant). Data represent the mean ± SD. (F) Growth curves of pbp1* pbp3 pbp4 in the presence or absence of IPTG. Data represent the mean ± SD. Error bars that are smaller than the data point symbols or have a negative value are not shown. Data are representative of two (A to D), three (E), and six (F) independent experiments. Download FIG S4, TIF file, 2.0 MB. Copyright © 2022 Wacnik et al. 2022 Wacnik et al. https://creativecommons.org/licenses/by/4.0/ This content is distributed under the terms of the Creative Commons Attribution 4.0 International license.

10.1128/mbio.00669-22.5 FIG S5 Gallery of AFM images of S. aureus Δ*pbp1*, *pbp1*_ΔPASTA_, and pbp1*. (A) Diagram of the section through of the cell with progressing septum (top) and AFM topographic images (bottom) of unfinished (i) and closed (ii) septa, parallel to the plane of the image, in SH1000 WT. Sacculi (images to the left, scale bars = 500 nm, data scales [*z*]: 200 [top] and 250 nm [bottom]) and higher-magnification scans (images to the right, scale bars = 50 nm, data scales [*z*]: 80 [top] and 40 nm [bottom]) of the boxed areas from the images to the left. (B) AFM topographic images of unfinished septa, parallel to the plane of the image, in Δ*pbp1* (from left to right, scale bars = 500, 50, and 50 nm; data scales [*z*] 500, 120, and 150 nm), *pbp1*_ΔPASTA_ (from left to right, scale bars = 500, 50, and 50 nm; data scales [*z*] 693, 80, and 100 nm), and pbp1* (from left to right, scale bars = 500, 50, and 50 nm; data scales [*z*] 500, 80, and 25 nm) grown in the absence of inducer for 2 h. Images to the left are sacculi, while images in the center (1) and to the right (2) are higher-magnification scans of the boxed areas of the images on the left. (C) AFM topographic images (right) of the external nascent ring architecture in SH1000 WT (*wt*; from top to bottom, scale bars = 500 and 50 nm; data scales [*z*], 100 and 20 nm) and mutants Δ*pbp1* (top to bottom, scale bars = 500 and 50 nm; data scales [*z*], 400 and 60 nm) and *pbp1*_ΔPASTA_ (from top to bottom, scale bars = 500 and 50 nm; data scales [*z*], 350 and 100 nm) grown in the absence of inducer for 2 h. The top images are the external surface of sacculi, while the bottom images are higher-magnification scans of the boxed areas of the top images. The arrows indicate piecrusts of the next division plane, which dissects the previous division septum. Arrowheads indicate abnormal features, holes, in the PG ring architecture. On the left is an interpretive diagram of a section through the cell wall (i) and the corresponding external surface (ii) as viewed by AFM. The mature cell wall of a newly separated daughter cell is shown in blue, which has both internally and externally mesh-structured PG. The newly exposed septum has an external ring-structured PG (green) and a mesh-like cytoplasmic facing PG (yellow). Data are representative of two independent experiments. Download FIG S5, TIF file, 1.3 MB. Copyright © 2022 Wacnik et al. 2022 Wacnik et al. https://creativecommons.org/licenses/by/4.0/ This content is distributed under the terms of the Creative Commons Attribution 4.0 International license.

### PBP1 TP activity is required for the characteristic septal PG architecture.

The pbp1* mutant gave a novel phenotype quite distinct from loss of entire PBP1 or PASTA domains. Inactivation of PBP1 TP activity did not prevent initiation and closing of the septa but, instead, resulted in accumulation of cells with aberrant septa and separation defects in about 80% of the population (Fig. 2A, C, and E). The septa in such cells had a rounded leading edge, were curved and abnormally thick (Fig. 2D and E and Fig. S4B and C), and had agglomerations of mesh-like material close to the septal center in addition to irregular piecrusts as observed by AFM (Fig. 2F and Fig. S5A and B). The intracellular agglomerations are PG, as they stain heavily with HADA and ADA-DA (Fig. 2A and Fig. S3C and F) and could be observed in purified sacculi (Fig. 2F and Fig. S5B). No ring architecture, only mesh-structured PG, could be observed on the surface of the pbp1* mutant. Importantly, with fluorescence microscopy, the pbp1* pbp3 pbp4 mutant, in which PBP2 is the only active TP, presented a similar phenotype upon IPTG removal as pbp1*, exemplified by misshapen septa and agglomerations of PG material marked by HADA (Fig. S4D). Therefore, aberrant septal synthesis and progression occur in the pbp1* mutant. Associated PG synthesis results from PBP2 transpeptidase activity and potentially the transglycosylase activity of FtsW, acting to produce un-cross-linked glycan strands.

**FIG 3:**
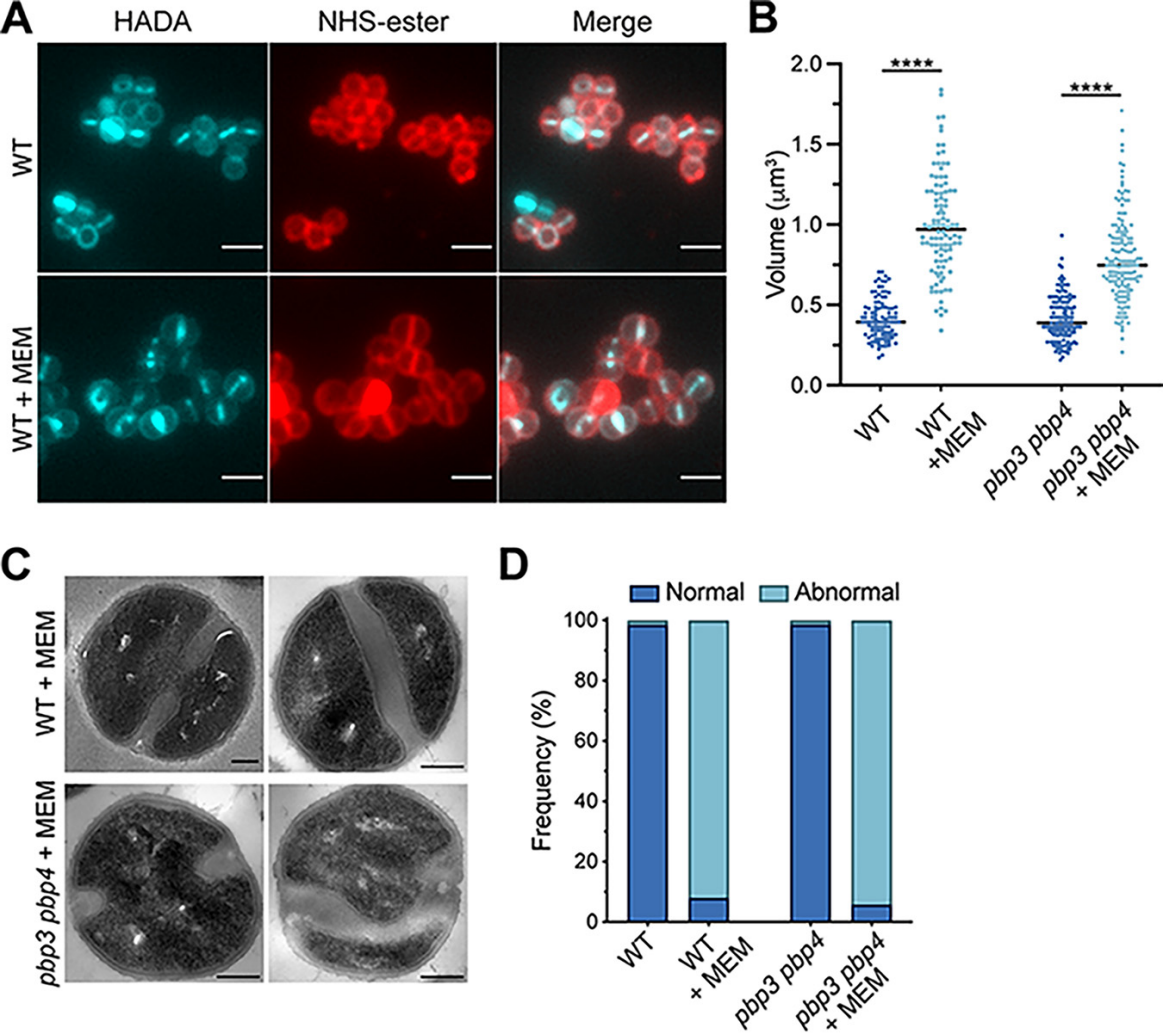
Effect of meropenem (MEM), an antibiotic with high affinity for PBP1, on S. aureus. (A) Fluorescence images of SH1000 WT treated with 1× MIC of MEM for 1 h, labeled with HADA for 5 min to show nascent PG, and counterlabeled with NHS-ester Alexa Fluor 555 (cell wall). Images are average intensity projections of z stacks. Scale bars = 2 μm. (B) Cell volumes of SH1000 WT and *pbp3 pbp4* treated with 1× MIC of MEM for 1 h as measured by fluorescence microscopy after NHS-ester Alexa Fluor 555 labeling (panel A). Each dot represents a single cell. The median of each distribution is indicated by a black line. The *P* value was determined by Mann-Whitney *U* tests (****, *P < *0.0001). From left to right, *P = *1.276e-042 and 1.303e-034. The number of cells analyzed for each condition was *n *≥ 100. (C) TEM of SH1000 WT and *pbp3 pbp4* treated with 1× MIC of MEM for 1 h. Scale bars = 200 nm. (D) Quantification of phenotypes of SH1000 WT and *pbp3 pbp4* treated with MEM (1× MIC) for 1 h based on TEM data (panel C and Fig. S6E and F). Examples of cells classified as normal (blue) are shown in Fig. S6E and F. Cells with abnormal phenotypes (light blue) are shown in panel C and Fig. S6E and F. From left to right, *n =* 343, 287, 403, and 365. Data are representative of two independent experiments.

The pbp1* phenotype occurred specifically because of the loss of the TP activity of this essential enzyme. This phenotype is mirrored by the mode of action of β-lactam antibiotics, which bind to and inhibit the TP activity of PBPs (7). We have recently described the morphological effects of methicillin and oxacillin on S. aureus, which result in cell swelling and cessation of septal and peripheral cell wall synthesis (31). Our results suggest that PBP1 TP activity has a role in septal plate formation, and without this, the septum is misshapen. The conditional lethal strains made here allow for functional analysis of the genes concerned. However, phenotypes tend to accumulate on depletion of the wild-type protein over time, confusing the precise roles for individual components. To independently corroborate the role of the TP activity of PBP1, we utilized an approach to directly, and selectively, inhibit its activity. Meropenem (MEM) has a higher affinity for PBP1 than PBP2 (32, 33), and therefore, we hypothesized that its effect on S. aureus would match that of pbp1*. In a MEM-titration, treatment with 1× MIC of MEM was sufficient to lead to cell death and a significant increase in SH1000 wild-type (WT) cell volume after 1 h (Fig. 3A and B and Fig. S6A). More than 70% of MEM-treated cells had growth defects that manifested as aberrantly shaped septa and accumulation of PG as shown by HADA labeling (Fig. 3A, C, and D and Fig. S6C and E), similar to observations made with the pbp1* mutants (Fig. 2A and C to E and Fig. S3C and F). The MEM phenotype of malformed septa was not linked to PBP3 or PBP4, as it was also observed in the corresponding double mutant (Fig. 3C and D and Fig. S6B, D, and F), which corroborated the role of PBP2 in misshapen septal genesis. The MEM phenotype differed from methicillin treatment, which inhibits both PBP1 and PBP2, as this results in a cessation of PG synthesis and apparent plasmolysis (31).

10.1128/mbio.00669-22.6 FIG S6 Effect of meropenem (MEM) on S. aureus. (A and B) Bactericidal effect of addition of 0.5×, 1×, 5×, and 10× MIC of MEM on (A) SH1000 WT and (B) *pbp3 pbp4*. The MEM MIC is 0.4 μg/mL and 0.2 μg/mL for SH1000 WT and *pbp3 pbp4*, respectively. Data represent the mean ± SD. Error bars that are smaller than the symbols are not shown. The dotted line is the detection limit. (C) Quantification of cellular phenotypes of SH1000 WT treated with 1× MIC of MEM for 1 h based on HADA incorporation (Fig. 3A). The same phenotype classification was used as that shown in Fig. 2C. From left to right, *n *= 309 and 355. (D) Fluorescence images of *pbp3 pbp4* treated with 1× MIC of MEM for 1 h, labeled with HADA for 5 min to show nascent PG and counterlabeled with NHS-ester Alexa Fluor 555 (cell wall). Images are average intensity projections of z stacks. Scale bars = 2 μm. Phenotype classification of MEM-treated *pbp3 pbp4* was not possible due to low HADA fluorescence signal. (E and F) TEM of SH1000 WT (E) and *pbp3 pbp4* (F) grown without (WT and *pbp3 pbp4*) or with 1× MIC of MEM (WT + MEM and *pbp3 pbp4 *+ MEM) for 1 h. Scale bars = 200 nm. Examples of cells categorized as normal phenotype are in blue; cells with abnormal phenotype are in light blue (1, asymmetric septum ingrowth; 2, off-septal PG thickening; 3, septum with rounded leading edge; 4, curved septum). Data are representative of three (A and B) and two (C to E) independent experiments. Download FIG S6, TIF file, 2.2 MB. Copyright © 2022 Wacnik et al. 2022 Wacnik et al. https://creativecommons.org/licenses/by/4.0/ This content is distributed under the terms of the Creative Commons Attribution 4.0 International license.

### PASTA domains mediate PBP1 interaction with division-associated components.

**FIG 4:**
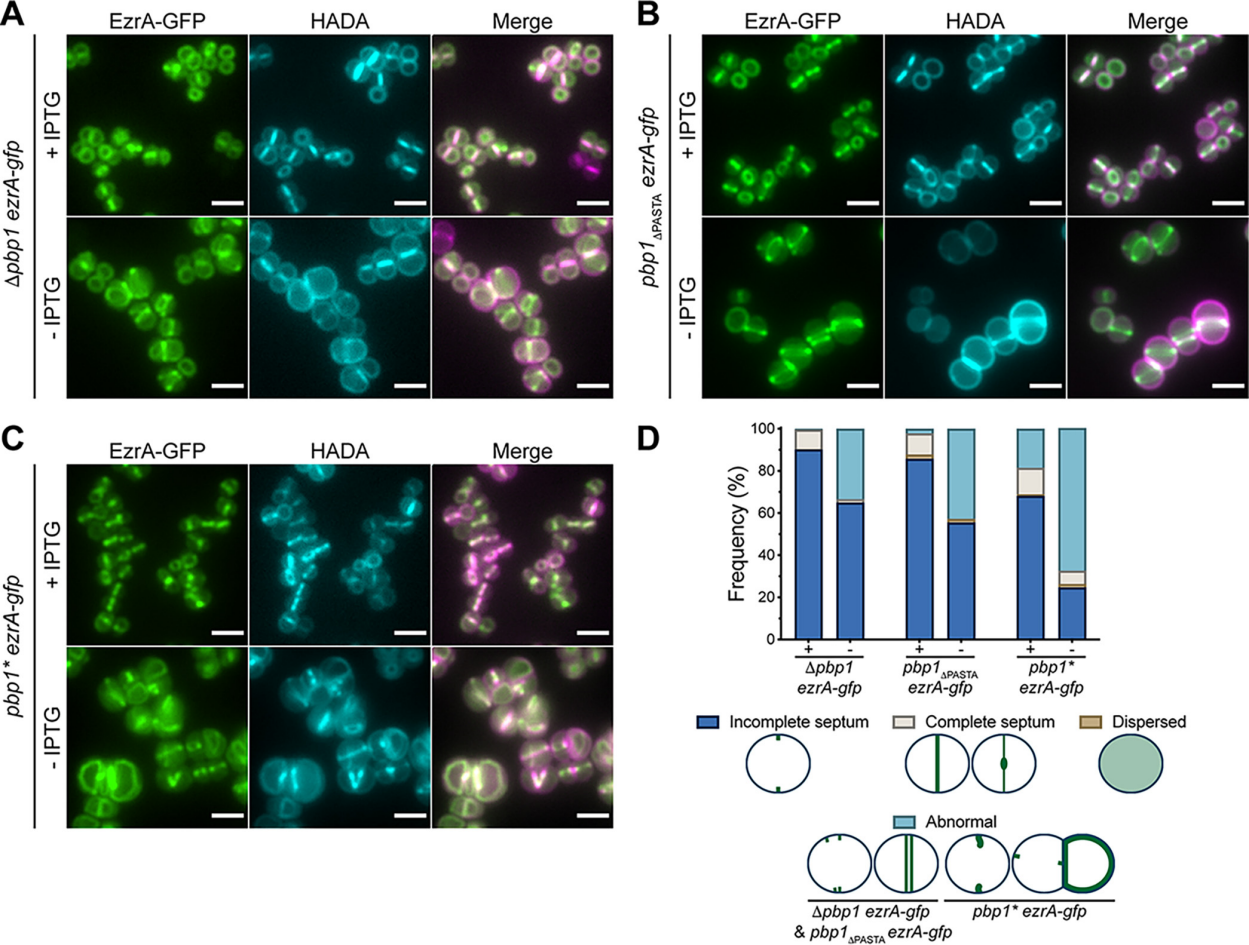
Role of PBP1, PASTA, and TP domains in EzrA localization in S. aureus. (A to C) Localization of EzrA-GFP in Δ*pbp1 ezrA-gfp*, *pbp1*_ΔPASTA_ *ezrA-gfp*, and pbp1* ezrA-gfp grown in the presence or absence of IPTG for 2 h and labeled with HADA for 5 min to stain PG. Images are average intensity projections of z stacks. Scale bars = 5 μm. (D) Quantification of EzrA-GFP localizations in Δ*pbp1 ezrA-gfp*, *pbp1*_ΔPASTA_ *ezrA-gfp*, and pbp1* ezrA-gfp grown with or without IPTG. “Abnormal” includes those cells with multiple and/or misplaced EzrA rings. From left to right, *n = *395, 499, 481, 438, 360, and 382. Data are representative of two independent experiments.

The morphologies of the Δ*pbp1* and *pbp1*_ΔPASTA_ mutants resemble S. aureus depleted of DivIB in which EzrA and FtsZ form multiple rings and the synthesis of the cross wall is blocked, despite the normal recruitment of early cell division proteins and piecrust formation (34). EzrA is a marker of early division protein recruitment but also remains until septal completion (9, 35). In the Δ*pbp1 ezrA-gfp* strain, EzrA, which here acts as an early cell division marker, was localized at midcell in the majority of cells and formed additional arcs or rings in 33% of the population (Fig. 4A and D). Multiple EzrA rings were observed in 43% of the *pbp1*_ΔPASTA_ *ezrA-gfp* mutant cells (Fig. 4B and D), supporting the requirement for PBP1 PASTA domains for correct selection of the division site and/or septal progression. Alternatively, the multiple division rings could result from a lack of the septal progression whereby the unproductive division machinery results in futile additional alternative initiation attempts, suggesting that PASTA domains are involved in the progression from piecrust to septal plate formation. While the number of cells with complete septa (EzrA-GFP visible as a line or focus) decreased by at least 6-fold in Δ*pbp1 ezrA-gfp* and *pbp1*_ΔPASTA_ *ezrA-gfp*, it only halved in pbp1* ezrA-gfp (12.5% to 6.3% of pbp1* ezrA-gfp grown with or without IPTG, respectively; Fig. 4C and D), confirming that septum progression, although reduced, still occurred when PBP1 TP was inactive, implying that TP activity is necessary for correct septal architecture during cell division.

PBP1 is important for the septal surface PG ring structure (Fig. 2F and Fig. S5B), where it has been proposed that mature WTA is not present throughout the septum (23, 36). Loss of WTA also results in a proportion of cells with aberrant septa (21), suggesting a potential link with PBP1 function. Loss of *tarO* (leading to a lack of WTA) caused minor cell division defects in SH1000 (Fig. S7A, E, and F). Combining *tarO* with the mutations in *pbp1* exacerbated the observed morphological defects, with the appearance of distinct septal and off-septal PG foci appearing (marked with HADA) in Δ*pbp1 tarO* and *pbp1*_ΔPASTA_ *tarO* (Fig. S7B to F), demonstrating that both WTA and PBP1 are involved in cell cycle progression in parallel.

10.1128/mbio.00669-22.7 FIG S7 Functional association between PBP1 and WTA. (A) Fluorescence images of the *tarO* mutant labeled with HADA for 5 min (nascent PG) and counterstained with NHS-ester Alexa Fluor 555 (cell wall). Images are average intensity projections of z stacks. Scale bars = 5 μm. (B to D) Δ*pbp1 tarO*, *pbp1*_ΔPASTA_ *tarO*, and pbp1* tarO grown with or without IPTG for 2 h, incubated with HADA for 5 min to show nascent PG, and counterlabeled with NHS-ester Alexa Fluor 555 (cell wall). Images are average intensity projections of z stacks. Scale bars = 5 μm. (D) Cell volumes of *tarO* and Δ*pbp1 tarO*, *pbp1*_ΔPASTA_ *tarO*, and pbp1* tarO grown with or without IPTG as measured by fluorescence microscopy after NHS-ester Alexa Fluor 555 labeling. Each dot represents a single cell. The median of each distribution is indicated by a black line. The *P* value was determined by Mann-Whitney *U* tests (***, *P < *0.0001). From left to right, *P = *2.243e-022, 1.460e-037, and 8.074e-029. The number of cells analyzed for each mutant and condition was *n *≥ 100. (F) Quantification of cellular phenotypes for *tarO* and Δ*pbp1 tarO*, *pbp1*_ΔPASTA_ *tarO*, and pbp1* tarO based on HADA incorporation (panels A to D) after incubation with or without IPTG. From left to right, *n *= 306, 253, 271, 358, 266, 313, and 336. Data are representative of two independent experiments. Download FIG S7, TIF file, 1.9 MB. Copyright © 2022 Wacnik et al. 2022 Wacnik et al. https://creativecommons.org/licenses/by/4.0/ This content is distributed under the terms of the Creative Commons Attribution 4.0 International license.

As PBP1 PASTA has a role in the regulation of septal plate formation, this may be determined by interacting with other protein components. In order to examine this hypothesis, we performed a bacterial two-hybrid assay, in which PBP1 has previously been found to have apparent, multiple interactions (35). Truncation of the PASTA domains reduced S. aureus PBP1 interaction not only with DivIB but also with FtsW, while recognition of other known interacting partners of PBP1 (EzrA, PBP2, and DivIC) were unaffected by the PASTA truncation (Fig. S8A and B), suggesting that these potential, wider interactions involve the N-terminal domain of PBP1.

10.1128/mbio.00669-22.8 FIG S8 The role of PBP1 PASTA domains in interactions with cell division components and PG. (A) Bacterial two-hybrid analysis of the effect of PASTA domain truncation on PBP1 interaction with its known interaction partners; empty, T18 with no insert; zip, T18 with a leucine zipper fragment; *ve*+, positive control (T18-zip/T25-zip); *ve*–, negative control (T18/T25). (B) Quantitative bacterial two-hybrid analysis of the effect of the PASTA domain truncation on PBP1 interaction with cell division components determined by analysis of the β-galactosidase activities of E. coli BTH101 cells harboring the corresponding plasmids. Dotted line, the positive interaction cutoff value (4-fold greater than the pair of T18/T25). Data represent the mean ± SD. The *P* value was determined by Mann-Whitney *U* tests (*, *P < *0.05). DivIB (PBP1 versus PBP1_ΔPASTA_) *P = *0.0424, FtsW (PBP1 versus PBP1_ΔPASTA_) *P = *0.0163. (C) Coomassie-stained SDS-PAGE gel (left) and BocillinFL gel-based analysis (right) of purified recombinant *Sa*PBP1, *Sa*BPP1*, *Sa*PBP1_ΔPASTA_, and *Sa*PASTA_PBP1_. Bands corresponding to *Sa*PBP1 and *Sa*PBP1_ΔPASTA_ were fluorescent, indicating their covalent binding to BocillinFL. Bands corresponding to *Sa*PBP1* and *Sa*PASTA_PBP1_ were not fluorescent and were therefore unable to bind BocillinFL. *Sa*PBP1 incubated with ampicillin prior to BocillinFL incubation failed to fluoresce, consistent with specific binding of BocillinFL to the TP domain. Expected sizes: *Sa*PBP1 and *Sa*PBP1*, 80.5 kDa; *Sa*PBP1_ΔPASTA_, 64.5 kDa; *Sa*PASTA_PBP1_, 18.2 kDa. (D) Cy2-labeled cytochrome *c* (Cy2-CytC) binding to purified peptidoglycan in the presence (+WTA) or absence (–WTA) of wall teichoic acid. Data represent the mean ± SD. Data are representative of two (C) and three (A and B) independent experiments. Download FIG S8, TIF file, 1.1 MB. Copyright © 2022 Wacnik et al. 2022 Wacnik et al. https://creativecommons.org/licenses/by/4.0/ This content is distributed under the terms of the Creative Commons Attribution 4.0 International license.

### PBP1 PASTA domains bind peptidoglycan.

**FIG 5:**
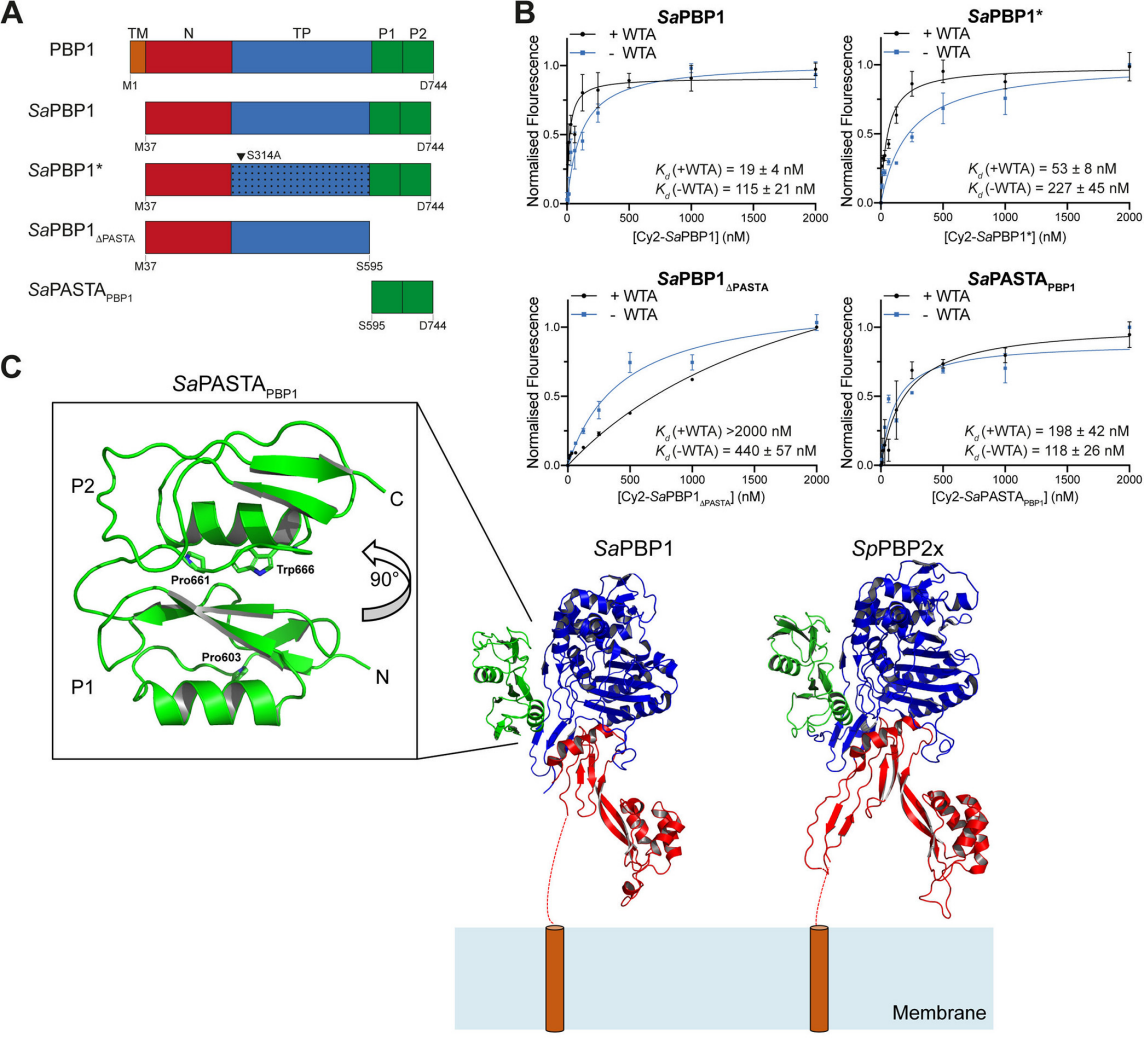
PBP1 PASTA domains bind cell walls. (A) Schematic representation of structural domain organization of S. aureus PBP1 (top) and recombinant proteins (*Sa*PBP1, *Sa*PBP1_ΔPASTA_, *Sa*PBP1*, and *Sa*PASTA_PBP1_) used in this study. TM, short cytoplasmic fragment and transmembrane helix (orange); N, N-terminal pedestal domain (red); TP, transpeptidase domain (blue); P, PASTA domains (green). The arrowhead indicates the inactivation substitution in the TP domain of *Sa*PBP1*. The first and last amino acids of constructs are indicated. (B) Fluorescence cell wall sedimentation assay. A Wilcoxon signed rank test (*P < *0.05) was carried out to assess the significance of difference in +WTA/–WTA PG binding: *Sa*PBP1, *P = *0.0273; *Sa*PBP1*, *P = *0.0078; *Sa*PBP1_ΔPASTA_, *P = *0.0195; *Sa*PASTA_PBP1_, *P ≥ *0.9999, not significant. Data represent the mean ± SD. Error bars that are smaller than the symbols are not shown. (C) Structure of *Sa*PASTA_PBP1_. The crystal structure of S. aureus PBP1 lacking the PASTA domains (*Sa*PBP1, PDB 5TRO) and our *Sa*PASTA_PBP1_ structure were superimposed on S. pneumoniae PBP2x (*Sp*PBP2x, PDB 5OAU) and displayed as cartoons. Their N termini are orientated close to the representative cell membrane, as if anchored there by their respective transmembrane helices (dashed red line/orange cylinder). Individual domains are colored as follows: N-terminal pedestal domain (red), transpeptidase domain (blue), and PASTA domain (green). The individual PASTA domains are labeled P1 and P2, respectively. Residues Pro603, Pro661, and Trp666 are displayed as sticks, with nitrogen atoms colored blue.

Impaired interaction with DivIB could be one explanation for why cells depleted of PBP1 PASTA domains initiate irregular piecrusts and septation defects accrue as a consequence. PASTA domains have long been associated with PG binding because of work performed mainly on serine/threonine protein kinases (STPK) (26, 37–39). Very recently, PBP1 PASTA domains have been shown to bind isolated, small fragments of PG (27). Therefore, we assessed whether S. aureus PBP1 and its PASTA domains could recognize PG by measuring their affinities for S. aureus cell wall PG with or without WTA (±WTA) with a semiquantitative fluorescence-binding assay and S. aureus PBP1 derivatives produced in Escherichia coli (Fig. 5A and Fig. S8C). Cytochrome *c* (34) was used as a negative control to rule our nonspecific binding (dissociation constant [*K_d_*], 1,126 ± 37 nM [+WTA] and 1,171 ± 363 nM [–WTA]) (Fig. S8D). Both *Sa*PBP1 (*K_d_*, 19 ± 4 nM [+WTA] and 115 ± 21 nM [–WTA]) and its PASTA domains (*Sa*PASTA_PBP1_; *K_d_*, 198 ± 42 nM [+WTA] and 109 ± 23 nM [–WTA]) bound PG (Fig. 5B). Inactive *Sa*PBP1* was still able to bind PG with a preference for PG with WTA present (*K_d_*, 53 ± 8 nM [+WTA] and 227 ± 46 nM [–WTA]; Fig. 5B), similar to active *Sa*PBP1. Although removal of the PASTA domains did not abolish BocillinFL binding (Fig. S8C), it considerably reduced the ability of *Sa*PBP1_ΔPASTA_ to bind PG, and binding was abolished in the presence of WTA (*K_d_*, >2,000 nM [+WTA] and 440 ± 57 nM [–WTA]; Fig. 5B). In contrast, the PASTA domains (*Sa*PASTA_PBP1_) on their own bind to S. aureus PG but are incapable of binding BocillinFL (Fig. 5B and Fig. S8C). These results demonstrate that PBP1 is a PG-binding protein, and the PASTA domains have a dominant role in this interaction. Sequence conservation analysis of PASTA domains revealed the presence of either Arg or Glu residues in classifying a PASTA domain as a PG-binder (40). The PASTA domains of S. aureus PBP1 each have proline at the equivalent positions (residues Pro603 and Pro661), and thus PBP1 would be predicted as a non-PG-binder. Our data suggest that the predicted significance of conserved Arg or Glu residues with regard to PG binding is either only relevant to PASTA domains found in STPKs, linear arrangements of tandem PASTA repeats, or is not suitable for proteins with multiple and complex functions like PBPs.

To gain a better understanding of the role of the PASTA domains in S. aureus PBP1 (*Sa*PASTA_PBP1_), we determined their structure by X-ray crystallography. Soluble recombinant protein was obtained in high yield from the cytoplasm of E. coli cells, and well-ordered crystals were subsequently produced that diffracted to a maximum resolution of 1.78 Å (Table S1). The structure was solved by molecular replacement using the corresponding PASTA domains present in *Sp*PBP2x from PDB entry 5OAU (41), which shares 26% sequence identity with *Sa*PASTA_PBP1_. The asymmetric unit contains two monomers (labeled A and B), each forming a 2-layer sandwich comprising an α-helix and a three-stranded antiparallel β-sheet, distinct from the TP domain (Fig. 5C). Clear and continuous electron density allowed the modeling and unambiguous assignment of both PASTA domains (Fig. 5C). When *Sa*PASTA_PBP1_ is compared with other structures deposited in the PDB using DALI (42), the top hit identified was S. pneumoniae PBP2x (Z-score, 15.7), showing a significant conservation of the PASTA fold despite low sequence identity (Fig. 5C). Unlike the linear arrangement observed for PASTA domains in serine/threonine kinases (43, 44), *Sa*PASTA_PBP1_ adopts a compact upside-down globular arrangement (Fig. 5C). The arrangement of the two PASTA domains solved here, in isolation from the TP domain in comparison to structural analyses of *Sp*PBP2x, is entirely consistent with a nonlinear PASTA domain arrangement. First, the structures of *Sa*PASTA_PBP1_ and the PASTA domains of *Sp*PBP2x share a pairwise root mean square deviation (RMSD) of 2.2 Å over 114 Cα, and when *Sa*PASTA_PBP1_ is superimposed on the PASTA domains of *Sp*PBP2x, there are no steric clashes with the TP domain. Second, the linker between PASTAs in *Sa*PASTA_PBP1_ has a sequence of DGDLTMPDMSGW, is neither glycine- nor alanine-rich, is not predicted to be disordered using the IUPred2 or ANCHOR2 web servers, and has a mean B factor of 44 Å^2^ in comparison to a mean B factor of 42 Å^2^ for the entire chain. Third, the interface between the PASTA domains is more reminiscent of the hydrophobic core of a globular protein than the more polar interface observed between molecules in crystal packing. Finally, the two proline residues that apparently define PBP1 as a nonbinder of PG are found buried from solvent either at the interface of PASTA domain 1 with the TP domain (Pro603) or at the interface between the TP domain and PASTA domains 1 and 2 (Pro661). The latter interface includes the only tryptophan (Trp666) in the sequence of *Sa*PASTA_PBP1_; tryptophan residues are frequent markers of carbohydrate binding sites in proteins (45), and in the absence of any obvious grooves or surface features associated with conserved sequence distributions and/or electrostatics, it remains unclear how the PASTA domains of *Sa*PBP1 recognize PG.

10.1128/mbio.00669-22.9 TABLE S1 Materials used in this study: (A) strains, (B) plasmids, (C) oligonucleotides, and (D) crystallographic data. Download Table S1, DOCX file, 0.05 MB. Copyright © 2022 Wacnik et al. 2022 Wacnik et al. https://creativecommons.org/licenses/by/4.0/ This content is distributed under the terms of the Creative Commons Attribution 4.0 International license.

## DISCUSSION

S. aureus has just two essential PBPs (46) and so forms an apparently simple system to understand cell wall growth and division. Even the transpeptidase activity of these two enzymes can be substituted by a single enzyme in the presence of β-lactam antibiotics via the acquisition of PBP2A, encoded by *mecA*, in MRSA strains. Our recent study has revealed that the presence of *mecA* and associated genetic lesions has a profound effect on S. aureus, even in the absence of antibiotics (25), leading to the discovery here that the PG biosynthetic activity of PBP1 is essential in MSSA but not in MRSA (Fig. 1D). This observation has important ramifications for many studies in S. aureus, where the use of an MRSA background can complicate phenotype interpretation. To understand the fundamental role of PBP1 activity in basic cell physiology, we have thus used an MSSA strain with a defined genetic background.

The essential function of PBP1 is associated with its crucial role in septal PG synthesis (14, 47). Here, we show that PBP1, in MSSA, has roles in both early and later stages of septum synthesis and can interact with other cell division components and make and bind to PG. PG binding is primarily mediated by the PASTA domains that are essential for cell division. There is clear overall structural similarity between S. aureus PBP1 and S. pneumoniae PBP2x PASTA domains in the way that the two tandem PASTA domains associate into an antiparallel bundle (Fig. 5C); this is in marked contrast to the head-to-tail linear PASTA domain repeats more typically found in STPKs. The highly hydrophobic interface between the two PASTA domains means it is unlikely to open up like butterfly wings to bind to PG; similarly, an extensive, linear interaction with PG, which is likely to occur with the head-to-tail PASTA domain arrangements seen in STPKs and which may require their dimerization (44), does not occur in *Sa*PBP1. Despite the successful production of diffracting crystals of *Sa*PASTA_PBP1_ grown in the presence of PG fragments (including an *N*-acetylglucosamine:*N*-acetylmuramic acid disaccharide), none of the structures yielded electron density features consistent with the stable binding of PG fragments. There are several potential explanations, including a lack of affinity of PASTA domains for small PG fragments, unrepresentative of the sacculus of S. aureus; our sedimentation assay does not permit the analysis of the binding of PASTA domains to small, soluble PG precursors. Consequently, and in common with all other PASTA domain structural analyses, the molecular details of PG recognition by *Sa*PBP1 remain elusive. During the preparation of the manuscript, Martínez-Caballero et al. (27) published a crystal structure of the two PASTA domains of PBP1, also in the absence of endogenous ligand, which is indistinguishable (RMSD, 0.7 Å over 204 superimposed residues) from the structure that we report here. The same authors also solved structures of *Sa*PBP1 in the presence and absence of β-lactams and pentaglycine (in which the PASTA domains were disordered). The latter structural analysis revealed that the pentaglycine substrate mimetic is not long enough to span between the transpeptidase active site and the PASTA domains, suggesting that the PG feature(s) recognized by the PASTA domain is/are chemically more complex than a simple short polypeptide.

**FIG 6:**
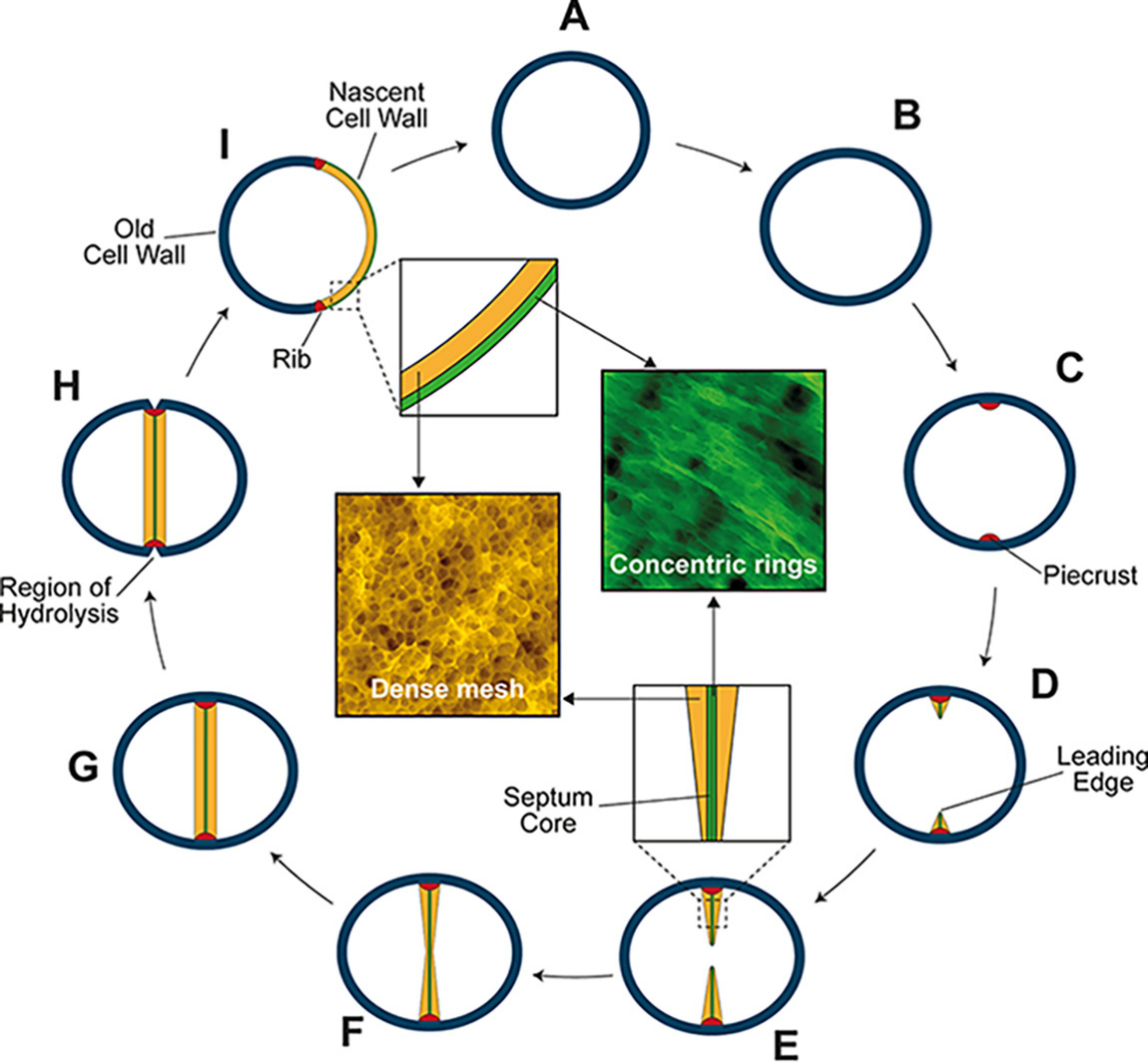
Conceptual model of septum formation in S. aureus, where PBP1 is required for septum formation and its characteristic ring-like PG architecture. (A and B) The growing S. aureus cell increases in volume (63). (C) Septal synthesis starts by formation of the piecrust (red) (30). This occurs as the result of the activity of PBP2 and forms the foundation for the septal plate. (D and E) The V-shaped septal plate (9) progresses inward by insertion of an initial, concentric ring-like structured PG synthesized by PBP1-FtsW at its core. Without PBP1 or PBP1 PASTA, the septal plate cannot be initiated, but in PBP1* it progresses but is aberrant. The PBP1-derived ring structure acts as a framework for the ensuing mesh-structured PG produced by PBP2. (F) The annulus closes, resulting in a bowed septum. (G) The septum is filled out by peptidoglycan insertion executed by PBP2, and this continues until the cross-wall is of uniform thickness (9). (H) The cell wall is hydrolyzed at the plane of septation. (I) Daughter cells separate. The cell wall of the daughter cell (colored insets) is a chimera of the old cell wall with both internally and externally mesh-structured PG and a nascent cell wall with the external ring-structured PG and the mesh-like cytoplasmic facing PG (5).

S. aureus is a spheroid coccus that can divide successively in orthogonal planes (30, 48). Septation is first observed as the formation of a band of PG known as the piecrust (30). This then transitions to the production of the septal plate itself, an initially V-shaped structure with a narrower leading edge (9). After closure of the septal annulus, the now bowed septum fills out to yield the mature structure prior to septal scission. The septal plate has two distinct PG architectures with a ring-like pattern at its core, which is exposed upon scission, and a subsequently synthesized fine mesh, akin to the rest of the peripheral cell wall (5). Loss of the entire PBP1, or just its PASTA domains, does not prevent piecrust formation but does result in multi- and/or off-center piecrusts without the ability to produce the septal plate (Fig. 2F). Thus, piecrust formation does not require PBP1 but is likely the result of the activity of the essential PBP2. PBP1 may regulate division site selection through PG cell wall recognition via its PASTA domains. Alternatively, as the division apparatus is unable to progress effectively to septal plate formation due to the lack of PBP1, this may lead to further rounds of initiation and piecrust formation. PBP1 has a clear role in septal plate formation where in the absence of PBP1 or the PASTA domains, cells form aberrantly shaped septa that do not close their annuli (Fig. 2A to E). In stark contrast, inactivation of PBP1 TP activity (pbp1*) does not stop inward septum progression, as observed with loss of PBP1 or the PASTA domains. However, such septa are misshapen, curved, and abnormally thick (Fig. 2A to E and Fig. 3). The use of the PBP1-specific antibiotic MEM at 1× MIC led to the similar morphology of thickened and misshapen septa. Two independent avenues of research both led to the conclusion that PBP1 TP activity is essential for continued division and colony formation, and while septum formation is disturbed, it is not entirely prevented. Therefore, PBP1 retains its regulatory function(s) regardless of activity loss. Loss of PBP1 activity may result in futile glycan strand synthesis (49) by its partner transglycosylase FtsW (14) and/or the continued activity of PBP2, resulting in the observed aberrant septa and stasis. FtsW in S. aureus is essential and required for septum progression (14). In Bacillus subtilis the cell division-associated PBP2B is essential, but its enzyme activity is not and can be compensated for by PBP3 (50). Here, in the *pbp3 pbp4* background loss of PBP1 activity did not lead to death of the cells (Fig. S4E and F), suggesting that the nonessential enzymes do not support survival in the absence of PBP1 TP activity, whereas deletion of the PASTA domains leads to rapid death of the cells (Fig. 1D) due to loss of protein functionality not observed in the pbp1*. Differences in plating efficiency and rate of loss of cellular viability between Δ*pbp1* and *pbp1*_ΔPASTA_ may reflect aberrant function of the truncated protein. As well as binding to the cell wall, PBP1 also apparently interacts with multiple protein partners, including EzrA, DivIB/C, PBP2, and FtsW (Fig. S8A and B) (14, 35). Recently, the PASTA domains from B. subtilis PBP2B were shown to regulate PBP2B interaction with DivIB (51). S. aureus DivIB is a PG-binding protein essential for division, the depletion of which leads to septal plate formation loss (34, 35). Here, the PBP1 PASTA domains were found to be involved in binding to DivIB and FtsW, alluding to their essential role in cell division. This could be a direct interaction, or loss of PBP1 PASTA may cause a conformational change in the remaining protein, as other's data suggest the FtsW-PBP1 interaction occurs via the PBP1 stalk domain (52). FtsW is a SEDS protein whose TG activity requires the presence of PBP1. Bifunctional aPBP (including PBP2) and bPBP-SEDS (including PBP1-FtsW) pairs share similar activities, but the fact that they coexist in many bacterial species implies there is a division of responsibilities between them. Indeed, it has been proposed lately that bPBP-SEDS pairs likely lay the primary PG matrix, while aPBPs support the initial PG by modifying, filling in, and adding PG to it (53, 54). The S. aureus septal plate PG has two distinct architectures, a disordered mesh present on its cytoplasm facing side and a ring structure at its core, which is revealed after the cells have split (5, 30) (Fig. 6). Recent AFM analysis from Staphylococcus warneri also describes the distinct PG architectures during septation as piecrust and septal plate rings/mesh (55). When sacculi are purified from *S. warneri*, the septum can split apart, revealing the rings, even in septa that have not closed their annulus, showing that the rings are not a likely result of PG hydrolysis during cell scission. We hypothesize (Fig. 6) that once the piecrust has been produced, PBP1 and FtsW use this as a foundation to initiate septal plate formation. Together they make the rings of material that become the core of the developing septum, providing the framework for PBP2 to make the bulk of the septal plate as a tight mesh alongside PBP4 and the insertion of WTA via the *tar* pathway. Loss of PBP1 TP activity in the presence of active PBP2 leads to the lack of the ring framework and aberrant, unproductive septum formation. The rings that form the center of the developing septum also provide the cleavage plane during scission.

Cell division is a fundamental requirement for life. A central question in bacteria is how is the division septum synthesized and then split to yield two daughter cells while maintaining cellular integrity in the face of internal turgor? Here, we have begun to answer this question by revealing the complex synthesis coordination mechanisms that allow this biological engineering feat to be accomplished.

## MATERIALS AND METHODS

### Bacterial growth conditions.

The strains used in this study are listed in Table S1A.

All Staphylococcus aureus strains were grown in tryptic soy broth (TSB) containing appropriate antibiotics at 37°C, unless otherwise indicated, with aeration. All Escherichia coli strains, unless otherwise stated, were grown in Lysogeny broth (LB) containing appropriate antibiotics at temperatures ranging from 20°C to 37°C with aeration. For solid medium, 1.5% (wt/vol) agar was added. When necessary, growth medium was supplemented with kanamycin (50 μg mL^−1^), tetracycline (1 μg mL^−1^), chloramphenicol (10 μg mL^−1^, S. aureus; 30 μg mL^−1^, E. coli), erythromycin (5 μg mL^−1^), spectinomycin (250 μg mL mL^−1^), ampicillin (100 μg mL^−1^), meropenem (0.4 μg mL^−1^, 1× MIC for SH1000 WT; 0.2 μg mL^−1^, 1× MIC for *pbp3 pbp4*), 5‐bromo‐4‐chloro‐3‐indolyl β‐d‐thiogalactopyranoside (X‐Gal; 80 μg mL^−1^, S. aureus; 40 μg mL^−1^, E. coli), or isopropyl β‐d‐thiogalactopyranoside (IPTG; 50 μM or 1 mM).

### Plasmid construction.

The plasmids and oligonucleotides used in this study are listed in Table S1 parts B and C, respectively.

Plasmids were cloned using E. coli NEB5α following previously described methods (56, 57).

### pKB-*Pspac-pbp1*.

A fragment containing the ribosome-binding site (RBS) and coding region of S. aureus *pbp1* was PCR amplified from the genomic DNA of S. aureus SH1000 using pCQ-pbp1-F/-R primers and cloned into pCQ11-FtsZ-SNAP (9) cut with NheI and AscI by Gibson assembly, resulting in pCQ11-P*spac*-*pbp1*. Next, the region containing P*spac*, RBS, and *pbp1* was PCR amplified from pCQ11-P*spac*-*pbp1* using pKB-Pspac-pbp1-F/-R primers and cloned into cloned into BamHI and EcoRI cut pKASBAR (34) by Gibson assembly, giving pKB-P*spac*-*pbp1*.

### pMAD-Δ*pbp1*.

Fragments encompassing 1-kb regions flanking upstream (from −980 bp upstream of *pbp1* to the first 20 bp of *pbp1*) and downstream (from 2,214 bp of *pbp1* to 970 bp downstream of *pbp1*) of *pbp1* were PCR amplified from S. aureus SH1000 genomic DNA using primer pairs pbp1-A/-B and pbp1-C/-D, respectively, and cloned into BamHI and EcoRI cut pMAD by Gibson assembly, creating deletion vector pMAD-Δ*pbp1.*

### pMAD-*pbp1*_ΔPASTA_.

Fragments encompassing 1.5-kb regions flanking the region encoding *pbp1* PASTA domains (upstream, from 286 bp to 1,785 bp of *pbp1*; downstream, from 2,214 bp of *pbp1* to 970 bp downstream of *pbp1*) were PCR amplified from S. aureus SH1000 genomic DNA using pbp1-E/-F and pbp1-G/-H primers and cloned into BamHI and EcoRI cut pMAD by Gibson assembly, resulting in deletion vector pMAD-*pbp1*_ΔPASTA_.

### pMAD-pbp1*.

An ~1.3-kb fragment covering an upstream region of the active site of *pbp1* (from −334 bp upstream of *pbp1* to the first 950 bp of the *pbp1* coding sequence) and an ~1.3-kb fragment comprising the 3′ fragment of *pbp1* (930- to 2,235-bp region of *pbp1*) were PCR amplified from S. aureus SH1000 genomic DNA using primer pairs pbp1*5′-F/-R and pbp1*3′-F/-R, respectively. Primers pbp1*5′-R and pbp1*3′-F were designed to introduce a T to G point mutation resulting in a Ser314Ala substitution. The PCR products were ligated with pMAD cut with EcoRI and BamHI by Gibson assembly, resulting in pMAD-pbp1*.

### T25-PBP1_ΔPASTA_.

A fragment carrying S. aureus *pbp1* without the PASTA domains (M1-S595) was PCR amplified from S. aureus SH1000 genomic DNA using T25-pbp1-F and T25-pbp1pasta-R and cloned into BamHI and EcoRI cut pKT25, resulting in T25-PBP1_ΔPASTA_.

### pVR plasmids.

Full-length *pbp1* (M1-D744) was E. coli codon optimized, synthesized with GenScript, PCR amplified using VR47F/R, and cloned into KpnI and HindIII cut pOPINRSF using In-Fusion cloning (TaKaRa Bio), resulting in pVR01. Construction of pVR02 (*Sa*PBP1, M37-D744) and pVR06 (*Sa*PASTA_PBP1_, S595-D744) was performed using inverse PCR (iPCR) (58), with pVR01 as a template and primer pairs VR49F/VR49R and VR57F/VR49R, respectively. pVR03 (*Sa*PBP1*, M37-D744, S314A) and pVR04 (*Sa*PBP1_ΔPASTA_, M37-S595) were constructed with QuikChange site-directed mutagenesis of pVR02 using VR51 and VR53, respectively.

### pSA50.

In order to construct an overexpression plasmid for sPBP1-BAP, the A51-D744 fragment of E. coli codon optimized *pbp1* was PCR amplified using primers OPPF20018F/OPPF20018R and cloned into KpnI and SfoI cut pOPINJB by In-Fusion cloning (TaKaRa Bio). The resulting construct, pSA50 contains an N-terminal hexahistidine tag fused to glutathione-S-transferase followed by a human rhinovirus 3C protease site, while the PBP1 (A51-D744) C-terminal end is fused to a biotin acceptor peptide (BAP) sequence.

### Construction of S. aureus mutants.

All vectors were passed through a restriction-deficient S. aureus RN4220 before being transduced into a final S. aureus SH1000 strain. Transformation and phage transduction of S. aureus were carried out as described previously (59, 60).

### Δ*pbp1*, *pbp1*_ΔPASTA_, and pbp1*.

For construction of *pbp1* mutation strains, first, an ectopic copy of *pbp1* under the control of the P*spac* promoter was introduced at the lipase (*geh*) locus. Electrocompetent CYL316 was transformed with pKB-P*spac*-*pbp1*. The chromosomal fragment containing the integrated plasmid was moved into S. aureus SH1000 by phage transduction, resulting in SJF4588 (S. aureus SH1000 *geh*::P*spac-pbp1*). Next, electrocompetent RN4220 was transformed with pMAD-Δ*pbp1*, pMAD-*pbp1*_ΔPASTA_, or pMAD-pbp1*, and the plasmids were moved to SJF4588 by phage transduction. Integration at 42°C and excision at 28°C of pMAD-Δ*pbp1*, pMAD-*pbp1*_ΔPASTA_, or pMAD-pbp1* resulted in strains SJF5116, SJF5275, and SJF4590, respectively. To allow controlled expression of *pbp1* from P*spac*, pGL485, a multicopy plasmid carrying *lacI* was introduced, creating strains Δ*pbp1* (S. aureus SH1000 *geh*::P*spac-pbp1* Δ*pbp1 lacI*), *pbp1*_ΔPASTA_ (S. aureus SH1000 *geh*::P*spac-pbp1 pbp1*_ΔPASTA_ *lacI*), and pbp1* (S. aureus SH1000 *geh*::Pspac-pbp1 pbp1* lacI). On all occasions, consistent colony size and growth kinetics were monitored to prevent the selection of suppressor mutations.

### MRSA Δ*pbp1* and MRSA pbp1*.

In order to construct high-level β-lactam-resistant mutants, Δ*pbp1* and pbp1* were transformed with a phage lysate from SJF5046 (S. aureus SH1000 *lysA*::p*mecA rpoB*^H929Q^) with selection for erythromycin resistance, resulting in low-level β-lactam-resistant Δ*pbp1* p*mecA* and pbp1* p*mecA.* The low-level-resistant mutants were transduced again with the phage lysate from SJF5046 and selected for kanamycin resistance, resulting in MRSA Δ*pbp1* (S. aureus SH1000 *geh*::P*spac-pbp1* Δ*pbp1 lacI lysA*::p*mecA rpoB*^H929Q^) and MRSA pbp1* pbp1 (S. aureus SH1000 *geh*::Pspac-pbp1 pbp1* lacI lysA::p*mecA rpoB*^H929Q^). MIC values were determined using antibiotic susceptibility tests using Etest MIC evaluator (Oxoid) strips.

### *pbp3 pbp4*.

SH1000 was transduced with a phage lysate from NE420 (S. aureus JE2 *pbp3*::*Tn*), resulting in SH4421 (S. aureus SH1000 *pbp3*::*Tn*). To swap the erythromycin resistance cassette to a kanamycin cassette, SH4425 (S. aureus SH1000 *pbp4*::*Tn*) was transduced with a phage lysate from NE3004 (S. aureus RN4220 pKAN). Integration at 42°C and excision at 28°C of pKAN resulted in strain SH5115 (S. aureus SH1000 *pbp4*::*kan*). SH4421 was subsequently transduced with a phage lysate from SH5115 (S. aureus SH1000 *pbp4*::*Tn*), resulting in *pbp3 pbp4* (SH5483; S. aureus SH1000 *pbp3*::*Tn pbp4*::*kan*).

### Δ*pbp1 pbp4*, *pbp1*_ΔPASTA_ *pbp4*, and pbp1* pbp4.

Δ*pbp1*, *pbp1*_ΔPASTA_, and pbp1* were transduced with a phage lysate from SH5115 (S. aureus SH1000 *pbp4*::*kan*), resulting in Δ*pbp1 pbp4* (S. aureus SH1000 *geh*::P*spac-pbp1* Δ*pbp1 lacI pbp4*::*kan*), *pbp1*_ΔPASTA_ *pbp4* (S. aureus SH1000 *geh*::P*spac-pbp1 pbp1*_ΔPASTA_ *lacI pbp4*::*kan*), and pbp1* pbp4 (S. aureus SH1000 *geh*::Pspac-pbp1 pbp1* lacI pbp4::*kan*), respectively.

### pbp1* pbp3 pbp4.

pbp1* pbp4 (S. aureus SH1000 *geh*::Pspac-pbp1 pbp1* lacI pbp4::*kan*) was transduced with a phage lysate from SH4421 (S. aureus SH1000 *pbp3*::*Tn*), resulting in pbp1* pbp3 pbp4 (S. aureus SH1000 *geh*::Pspac-pbp1 pbp1* lacI pbp3::*Tn pbp4*::*kan*).

### Δ*pbp1 ezrA-gfp*, *pbp1*_ΔPASTA_ *ezrA-gfp*, and pbp1* *ezrA-gfp*.

Δ*pbp1*, *pbp1*_ΔPASTA_, and pbp1* were transduced with a phage lysate from JGL227 (S. aureus SH1000 *ezrA-gfp+*) (35), resulting in Δ*pbp1 ezrA-gfp* (S. aureus SH1000 *geh*::P*spac-pbp1* Δ*pbp1 lacI ezrA-gfp*), *pbp1*_ΔPASTA_ *ezrA-gfp* (S. aureus SH1000 *geh*::P*spac-pbp1 pbp1*_ΔPASTA_ *lacI ezrA-gfp*), and pbp1* ezrA-gfp (S. aureus SH1000 *geh*::Pspac-pbp1 pbp1* lacI ezrA-gfp), respectively.

### Δ*pbp1 tarO*, *pbp1*_ΔPASTA_ *tarO*, and pbp1* tarO.

Δ*pbp1*, *pbp1*_ΔPASTA_, and pbp1* were transduced with a phage lysate from *tarO* (S. aureus SA113 Δ*tarO*::*ery* pUC1-*tarO*) (31), resulting in Δ*pbp1 tarO* (S. aureus SH1000 *geh*::P*spac-pbp1* Δ*pbp1 lacI* Δ*tarO*::*ery*), *pbp1*_ΔPASTA_ *tarO* (S. aureus SH1000 *geh*::P*spac-pbp1 pbp1*_ΔPASTA_ *lacI* Δ*tarO*::*ery*), and pbp1* tarO (S. aureus SH1000 *geh*::Pspac-pbp1 pbp1* lacI Δ*tarO*::*ery*), respectively.

### PBP1 depletion.

P*spac-pbp1* strains were grown from an optical density at 600 nm (OD_600_) of 0.1 to the exponential phase (OD_600_ ~0.5) in TSB containing 10 μg mL^−1^ chloramphenicol and 50 μM IPTG. Cells were washed three times by centrifugation and resuspension in TSB. Washed cells were then used to inoculate TSB containing 10 μg mL^−1^ chloramphenicol. Cultures were inoculated to an OD_600_ of 0.05 for phenotypic studies and an OD_600_ of 0.005 for growth studies. For phenotypic analysis, cultures were incubated for 2 h to allow depletion of PBP1 before microscopy imaging. Control samples were grown in TSB supplemented with 10 μg mL^−1^ chloramphenicol and 1 mM (50 μM, *ezrA-gfp* mutants) IPTG.

For the plating efficiency test, cells grown in the presence of 10 μg mL^−1^ chloramphenicol and 50 μM IPTG to the exponential phase (OD_600_, ~0.5) were washed three times in phosphate-buffered saline (PBS). Serial dilutions of washed cells were plated on TSB containing 10 μg mL^−1^ chloramphenicol, with or without 1 mM IPTG. Relative plating efficiency (% CFU) is expressed as the number of cells that grow on plates without IPTG (CFU*_no IPTG_*) to cells that grow in the presence of IPTG (CFU*_IPTG_*) multiplied by 100%:

$$\text{\%}\mathrm{CFU}=\frac{\mathrm{CF}U_{\mathrm{no}\;\mathrm{IPTG}}}{\mathrm{CF}U_{\mathrm{IPTG}}}\times 100\text{\%}$$

### Meropenem activity assays.

S. aureus strains were grown overnight in TSB. The overnight cultures were used to inoculate fresh TSB medium to an OD_600_ of 0.05. When cells reached an OD_600_ of 0.2 to 0.4, meropenem was added, and the change of bacterial count was monitored. The CFU per mL of culture measures were normalized to the initial CFU/mL at the time of the antibiotic addition, at time zero (*t_0_*).

$$\mathrm{Relative}\;\mathrm{CFU}/\mathrm{mL}=\frac{\mathrm{CFU}/mL_{\mathrm{tn}}}{\mathrm{CFU}/mL_{t0}}$$

For phenotypic analysis, cells were treated for 1 h with 1× MIC meropenem before microscopy imaging.

### Fractionation of S. aureus membranes.

The membrane fraction of S. aureus was prepared as previously described (61) with the following modifications. S. aureus cells grown to the appropriate growth phase were recovered by centrifugation (5,000 × *g*, 10 min, 4°C) and washed three times by resuspension and centrifugation (5,000 × *g*, 10 min, 4°C) in PBS. Cells were resuspended in 50 mM Tris, 100 mM NaCl, pH 8.0 containing Complete Protease Inhibitor (Roche) and broken using 0.1-mm silica spheres (lysing matrix B) and FastPrep homogenizer (MP Biomedicals) in 12 cycles of 30 s, at maximum speed (6.5 m s^−1^), with 5 min of incubation on ice between cycles. Cell lysates were centrifuged (8,000 × *g*, 10 min, 4°C) to remove unbroken cells. The supernatant was then spun (8,000 × *g*, 10 min, 4°C) to sediment cell wall material. The membrane fraction was recovered from the supernatant by centrifugation (35,000 × *g*, 20 min, 4°C), and the pellet (membranes) was resuspended in PBS. The total protein concentration was estimated by Bradford assay.

### *In vitro* labeling of S. aureus PBPs with BocillinFL.

This method was adopted from a published protocol (62) with minor modifications. Membrane proteome samples (25 μg in 20 μL PBS) and purified proteins (2.5 μg in 25 μL HEPES pH 7.5 150 mM NaCl) were incubated with 25 μM BocillinFL (Invitrogen) for 20 min at 37°C. Additionally, for the competition assay, purified *Sa*PBP1 was mixed with 2.5 μg (~286 μM final concentration) ampicillin and incubated at 37°C for 10 min prior to the addition of BocillinFL. The reaction was stopped by the addition of 5× SDS-PAGE loading buffer. Membrane proteome was additionally incubated for 10 min at 90°C. The samples were run on a 6 to 20% (wt/vol) SDS-PAGE gradient or 10% (wt/vol) SDS-PAGE gel and visualized using a Bio-Rad ChemiDoc MP imaging system or a GE Typhoon FLA 9500.

### Labeling S. aureus d-amino acids.

S. aureus cells were incubated with 500 μM (2 mM for *pbp4* mutants) HADA or 1 mM ADA-DA at 37°C for 5 min. Cells were then washed by centrifugation and resuspension in PBS.

### Click chemistry.

ADA-DA containing an azide functional group was fluorescently labeled with Atto 488 alkyne at 5 μg mL^−1^ via the click reaction [copper (I)-catalyzed alkyne-azide cycloaddition]. This was carried out using the Click-iT cell reaction buffer kit (Thermo Fisher) according to the manufacturer’s protocol.

### Labeling S. aureus with fluorescent NHS-ester.

Fixed cell wells were resuspended in PBS containing 8 μg mL^−1^ Alexa Fluor 555 NHS-ester (Invitrogen) and incubated at room temperature for 30 min. Cells were washed twice by centrifugation and resuspension in PBS.

### Fixing for fluorescence microscopy.

Cells were fixed by incubation in 1.6% (wt/vol) paraformaldehyde at room temperature for 30 min.

### Fluorescence microscopy.

Fixed cells were dried onto a poly l-lysine-coated slide, mounted in PBS, and imaged on a Nikon Ti inverted microscope fitted with a Lumencor Spectra X light engine. Images were taken using a 100× PlanApo (1.4 NA) oil objective using 1.518 RI oil and detected by an Andor Zyla sCMOS camera.

### Cell volume estimation.

Cell volume calculations were carried out as previously described (63). The long and short axes of cells were measured using Fiji. The volume was then calculated based on a prolate spheroid shape with volume

$$V=4/3\pi ab^{2},$$

where *a* and *b* are the radii along the long and short axes, respectively.

### Transmission electron microscopy.

S. aureus strains were prepared for electron microscopy as previously described (64).

### Preparation of S. aureus sacculi.

Peptidoglycan from S. aureus cells was extracted and, if required, hydrofluoric acid (HF)-treated to remove cell wall accessory polymers as previously described (64).

### Sacculi immobilization for AFM imaging.

The immobilization surface was prepared by adding the solution mixed with 171 μL of 100 mM NaHCO_3_, 3 μL of 1 M NaOH, and 6 μL of Cell-Tak (Corning, 5% [wt/vol] in acetic acid) on freshly cleaved mica. After 30 min of incubation, the surface was washed with 5 × 200 μL high-pressure liquid chromatography (HPLC)-grade water. Sacculi stocks were 10 times diluted in HPLC-grade water and briefly tip-sonicated to resuspend them prior to immobilization. Then, 10 μL of the sacculi suspension was added to 40 μL of HPLC-grade water on the Cell-Tak immobilization surface and incubated for 1 h. The surface was then thoroughly rinsed with HPLC-grade water, blow-dried with nitrogen, and stored in a petri dish at room temperature before AFM imaging.

### AFM Imaging and image analysis.

AFM imaging was carried out on a Nanowizard III ULTRA Speed system (JPK, Germany). Rectangular cantilevers with a nominal spring constant of 0.3 N/m and resonant frequency (in liquid) of ~150 kHz (USC-F0.3-k0.3; NanoWorld, Switzerland) were used. The spring constant and deflection sensitivity of each cantilever were calibrated prior to each measurement (65, 66) Measurements were carried out in quantitative imaging mode at room temperature in the buffer composed of 200 mM KCl and 10 mM Tris. Scans were driven at a line rate of ~0.78 Hz, with a typical Z length of 300 nm and trigger force of 20 nN. The resultant topographic images were processed using JPK Data Processing. No flattening or surface subtraction was applied. A high-pass filter (scale: 100% to 500%, degree of smoothing: 5 px, horizontal) was applied to the higher magnification images to enhance the contrast without modifying the morphological features. The morphological features of sacculi were summarized from images obtained on abundant technical repeats of 2 biological replicates.

### Recombinant protein production and purification.

**sPBP1-BAP.** E. coli BL21(DE3) cells containing plasmid pSA50 were grown in LB medium supplemented with 100 μg mL^−1^ ampicillin at 37°C to an OD_578_ of 0.5. Protein overproduction was induced by addition of 0.5 mM IPTG to the cell culture and further incubation for 4 h at 30°C. Cells were harvested by centrifugation (6,200 × *g*, 15 min, 4°C), and the pellet was resuspended in basic buffer (25 mM Tris-HCl, 100 mM NaCl, pH 7.5). After addition of 1 mM phenylmethylsulfonyl fluoride (PMSF), a 1:1,000 dilution of protease inhibitor cocktail (Sigma-Aldrich), and DNase, the cells were disrupted by sonication (Branson digital sonifier). The cell lysate was centrifuged (130,000 × *g*, 60 min, 4°C), and the supernatant was recovered. The supernatant was incubated with Ni-NTA Superflow (Qiagen) for 2 h at 4°C with gentle stirring, which had been preequilibrated in basic buffer. The resin was poured into a gravity column and washed with 20 volumes of wash buffer (25 mM Tris-HCl, 150 mM NaCl, 10% [vol/vol] glycerol, 10 mM MgCl_2_, 20 mM imidazole, pH 7.5). Bound protein was eluted with elution buffer (25 mM Tris-HCl, 150 mM NaCl, 10% [vol/vol] glycerol, 10 mM MgCl_2_, 600 mM imidazole, pH 7.5). Next, 10 U mL^−1^ of HRV-3C protease (TaKaRa) was added to the Ni-NTA-eluted protein to remove the oligohistidine-GST tag during dialysis against 3 L of dialysis buffer I (25 mM Tris-HCl, 150 mM NaCl, 10 mM EGTA, 10% [vol/vol] glycerol, pH 7.5) for 20 h at 4°C. Digested protein was dialyzed against 3 L of dialysis buffer II (25 mM Tris-HCl, 150 mM NaCl, 10 mM MgCl_2_, 10% [vol/vol] glycerol, pH 7.5), for 3 h at 4°C. The protein was incubated in the same Ni-NTA beads (preequilibrated in dialysis buffer II) for 2 h at 4°C to remove the contaminants and the His-GST tag from the sample. The flowthrough and the washes (2 volumes of wash buffer) were pooled, dialyzed against storage buffer (25 mM HEPES-NaOH, 150 mM NaCl, 10 mM MgCl_2_, 10% [vol/vol] glycerol, pH 7.5), and concentrated using a Vivaspin Turbo 15 column (molecular weight cutoff [MWCO] of 50,000 Da).

### *Sa*PBP1, *Sa*PBP1*, *Sa*PBP1_ΔPASTA_, and *Sa*PASTA_PBP1_.

All recombinant proteins were produced in E. coli Rosetta (DE3) cells at 37°C in Terrific Broth (TB) medium supplemented with 50 μg mL^−1^ kanamycin and 30 μg mL^−1^ chloramphenicol. Once cultures had reached an OD_600_ of 0.9, protein expression was induced with 1 mM IPTG for 20 h at 20°C. Cells were harvested by centrifugation (4,000 × *g* at 4°C for 30 min), and the pellet was resuspended in a buffer of 50 mM Tris-HCl, pH 8.0, 500 mM NaCl, 20 mM imidazole supplemented with one EDTA-free protease inhibitor cocktail tablet (Roche), and DNase (4 μg mL^−1^ final concentration). The cells in this resuspension were lysed by two passes through a One-Shot cell disruptor (Constant Systems) at 23 kilopounds per square inch (kpsi), and the cell debris was removed by centrifugation (40,000 × *g* at 4°C for 30 min). The first purification step was affinity chromatography with a 5-mL HisTrap FF column (GE Healthcare) precharged with Ni^2+^ and equilibrated in buffer A (50 mM Tris-HCl, pH 8.0, 500 mM NaCl, 20 mM imidazole). A linear concentration gradient of imidazole was applied to elute the protein using buffer B (50 mM Tris-HCl, pH 8.0, 500 mM NaCl, 800 mM imidazole). Further purification was carried out by size exclusion chromatography using a Superdex 200 Hi Load 16/60 column (GE Healthcare). Proteins were eluted with SEC buffer (25 mM Tris-HCl, pH 8.0, 150 mM NaCl) and analyzed by SDS-PAGE.

### Generation of anti-PBP1 antibody.

Serum against sPBP1A-BAP was produced from rabbits following a 28-day immunization program at Eurogentec (Belgium), and it was purified as previously described (67).

### Immunoblot analysis.

S. aureus cultures were washed three times by resuspension and centrifugation (5,000 × *g*, 10 min, 4°C) in PBS. Cells were resuspended in TBSI (50 mM Tris, 100 mM NaCl, pH 8.0, plus Complete Protease Inhibitor Cocktail [Roche]) and broken using 0.1 mm silica spheres (lysing matrix B) and FastPrep homogenizer (MP Biomedicals) in 12 cycles of 30 s, at maximum speed (6.5 m s^−1^), with a 5-min incubation on ice between cycles. Cell lysates were centrifuged (5,000 × *g*, 10 min, 4°C) to remove unbroken cells. Approximately 60 μg of total protein was separated on a 12% (wt/vol) SDS-PAGE gel and electroblotted onto a nitrocellulose membrane and blocked in 5% (wt/vol) skimmed milk in TBST (20 mM Tris-HCl, pH 7.6, 17 mM NaCl, 0.1% [vol/vol] Tween 20). The membrane was incubated with primary polyclonal anti-PBP1 (1:1,000) overnight with gentle agitation at 4°C. Primary antibodies were detected using horseradish peroxidase-conjugated goat anti-rabbit IgG (1:10,000; Bio-Rad) and Clarity Western enhanced chemiluminescence (ECL) substrate (Bio-Rad) reagent according to the manufacturer’s protocol. Chemiluminescence was detected using a Syngene G:BOX Chemi XX9 system.

### Crystallization, data collection, and structure determination.

Crystallization of *Sa*PASTA_PBP1_ was carried out at 20°C by the sitting-drop vapor diffusion method in 96-well MRC plates (Molecular Dimensions) with a Mosquito crystallization robot (TTP LabTech) and commercial crystallization screens (Hampton Research and Molecular Dimensions). Orthorhombic crystals of diffraction quality, with a maximum dimension of approximately 500 μm, appeared overnight from a mixture of equal volumes of protein solution (42 mg mL^−1^ in 25 mM Tris-HCl, pH 8.0, 150 mM NaCl) and reservoir solution (0.2 M NaCl, 0.1 M sodium/potassium phosphate, pH 6.2, 50% [vol/vol] polyethylene glycol [PEG] 200). Diffraction data were indexed and integrated using XDS (68) and scaled using AIMLESS (69) from the CCP4 program suite (70). The crystals displayed space group *P*2_1_2_1_2_1_ with unit cell lengths *a *= 39.8 Å, *b *= 81.4 Å, and *c *= 89.6 Å. The asymmetric unit consisted of two polypeptide chains with an estimated solvent content of 45% and a V_m_ (Matthew's coefficient) of 2.24 Å^3^/Da. The region corresponding to the two PASTA domains in the crystal structure of S. pneumoniae PBP2x (PBP 5OAU) was used as a molecular replacement search model, sharing approximately 26% sequence identity with *Sa*PASTA_PBP1_. The search model was generated using phenix.sculptor (71) to remove nonmacromolecular chains and prune sidechains. The structure was solved by molecular replacement using PHASER (72), and the resultant electron density map was of high quality, allowing the tracing of the main chain. Model building and refinement were carried out with Coot (73) and Phenix (74), respectively. Assessments of the geometry and validation of the final model were carried out using MolProbity (75). Analyses of surface areas and interactions were made using the PISA web service (76). The graphics program PyMOL (Schrӧdinger, LLC) was used to generate all molecular figures presented.

### Cell wall binding assays.

Cell wall binding assays of recombinant PBP1 proteins fluorescently labeled with Cy2 bis‐reactive dye (GE Healthcare) were performed as previously described (34), except for the binding buffer: 25 mM HEPES (pH 7.5), 150 mM NaCl, and 10 mM MgCl_2_. Cy2-labeled cytochrome *c* (Cy2-CytC) was included as a negative control. Binding of fluorescently labeled proteins was determined by fluorescence measurements using a Hidex Sense plate reader (excitation at 490/20 nm and emission at 535/20 nm).

### Bacterial two-hybrid assay.

Competent BTH101 was cotransformed with pKT25 and pUT18 derivatives. Transformants were selected on LB agar plates containing 100 μg mL^−1^ ampicillin, 50 μg mL^−1^ kanamycin, and 40 μg mL^−1^ X‐Gal and incubated at 30°C. Single colonies were grown in 150 μL LB with 100 μg mL^−1^ ampicillin, 50 μg mL^−1^ kanamycin, and 0.5 mM IPTG at 30°C.

To qualitatively measure for pairwise interactions, 5 μL of each overnight culture was spotted onto LB agar plates containing 100 μg mL^−1^ ampicillin, 50 μg mL^−1^ kanamycin, 0.5 mM IPTG, and 40 μg mL^−1^ X‐Gal. The plates were incubated at 30°C for 24 to 48 h in an environment protected from light and imaged. To quantify interactions, overnight cultures were assayed for β‐galactosidase activity against MUG (4‐methylumbelliferyl‐β‐d‐galactopyranoside) using an assay as previously described (35).

### Data availability.

All study data are included in the article and/or supporting information. The data that support the findings of this study are available in the Online Research Data figshare from the University of Sheffield with the identifier https://figshare.shef.ac.uk/collections/Penicillin-Binding_Protein_1_PBP1_of_Staphylococcus_aureus_Has_Multiple_Essential_Functions_in_Cell_Division/5656339/1. The crystal structure of the S. aureus PBP1 PASTA domains (PDB ID 7O61) can be accessed at https://www.rcsb.org/structure/7O61.
