# Supplementary material for: Penicillin-Binding Protein 1 (PBP1) of Staphylococcus aureus Has Multiple Essential Functions in Cell Division
Source: mBio. 2022 Jun 15;13(4):e00669-22. doi: 10.1128/mbio.00669-22 (PMC9426605; doi:10.1128/mbio.00669-22)
Supplement: TABLE S1 [file mbio.00669-22-s0001.docx]

**Supplementary Table 1**

**Table S1A. Strains used in this study**

| **Name** | **Relevant genotype/Markers** | **Source** |
| --- | --- | --- |
| *Staphylococcus aureus* | | |
| SH1000 | Functional *rsbU*+ derivative of *S. aureus* 8325-4 | (1) |
| VF17 | SH1000 pGL485 (*lacI*); Cm^R^ | (2) |
| RN4220 | Restriction deficient transformation recipient | (3) |
| CYL316 | RN4220 pCL112Δ19 | (4) |
| SJF4588 | SH1000 *geh::*P*spac-pbp1*; Tet^R^ | This study |
| SJF5116 | SH1000 *geh::*P*spac* *-pbp1* Δ*pbp1*; Tet^R^ | This study |
| SJF5275 | SH1000 *geh::*P*spac* *-pbp1 pbp1*_ΔPASTA_; Tet^R^ | This study |
| SJF4590 | SH1000 *geh::*P*spac* *-pbp1 pbp1** Tet^R^, Cm^R^ | This study |
| Δ*pbp1* | SH1000 *geh::*P*spac* *-pbp1* Δ*pbp1 lacI*; Tet^R^ | This study |
| *pbp1*_ΔPASTA_ | SH1000 *geh::*P*spac* *-pbp1 pbp1*_ΔPASTA_ *lacI*; Tet^R^, Cm^R^ | This study |
| *pbp1** | SH1000 *geh::*P*spac* *-pbp1 pbp1* lacI*; Tet^R^, Cm^R^ | This study |
| SJF5046 | SH1000 *lysA::*p*mecA rpoB*^H929Q^; Ery^R^, Kan^R^ | (5) |
| Δ*pbp1* p*mecA* | SH1000 *geh::*P*spac-pbp1* Δ*pbp1 lacI lysA::*p*mecA*; Tet^R^, Cm^R^, Ery^R^ | This study |
| *pbp1** p*mecA* | SH1000 *geh::*P*spac-pbp1 pbp1* lacI lysA::*p*mecA*; Tet^R^, Cm^R^, Ery^R^ | This study |
| MRSA Δ*pbp1* | SH1000 *geh::*P*spac-pbp1* Δ*pbp1 lacI lysA::*p*mecA rpoB*^H929Q^; Tet^R^, Cm^R^, Ery^R^, Kan^R^ | This study |
| MRSA *pbp1** | SH1000 *geh::*P*spac-pbp1 pbp1* lacI lysA::*p*mecA rpoB*^H929Q^; Tet^R^, Cm^R^, Ery^R^, Kan^R^ | This study |
| JGL227 | SH1000 *ezrA-gfp+*; Ery^R^ | (2) |
| Δ*pbp1 ezrA-gfp* | SH1000 *geh::*P*spac-pbp1* Δ*pbp1 lacI ezrA-gfp*; Tet^R^, Cm^R^, Ery^R^ | This study |
| *pbp1*_ΔPASTA_ *ezrA-gfp* | SH1000 *geh::*P*spac-pbp1 pbp1*_ΔPASTA_ *lacI ezrA-gfp*; Tet^R^, Cm^R^, Ery^R^ | This study |
| *pbp1* ezrA-gfp* | SH1000 *geh::*P*spac-pbp1 pbp1* lacI ezrA-gfp*; Tet^R^, Cm^R^, Ery^R^ | This study |
| NE420 | JE2 *pbp3::Tn*; Ery^R^ | (6) |
| SH4421 | SH1000 *pbp3::Tn*; Ery^R^ | This study |
| NE3004 | RN4220 pKAN; Cm^R^, Ery^R^ | (6) |
| SH4425 | *pbp4::Tn*; Ery^R^ | (7) |
| SH5115 | SH1000 *pbp4::kan*; Kan^R^ | This study |
| *pbp3 pbp4* (SH5483) | *pbp3::Tn pbp4::kan*; Ery^R^ *,*Kan^R^, | This study |
| Δ*pbp1 pbp4* | SH1000 *geh::*P*spac-pbp1* Δ*pbp1 lacI pbp4::Tn*; Tet^R^, Cm^R^, Kan^R^ | This study |
| *pbp1*_ΔPASTA_ *pbp4* | SH1000 *geh::*P*spac-pbp1 pbp1*_ΔPASTA_ *lacI pbp4::Tn*; Tet^R^, Cm^R^, Kan^R^ | This study |
| *pbp1* pbp4* | SH1000 *geh::*P*spac-pbp1 pbp1* lacI pbp4::Tn*; Tet^R^, Cm^R^, Kan^R^ | This study |
| *tarO* | SH1000 Δ*tarO::ery*; Ery^R^ | (8) |
| *tarO tarO+* | SA113 Δ*tarO::ery* pUC1-*tarO* ; Ery^R^, Cm^R^ | (8) |
| Δ*pbp1 tarO* | SH1000 *geh::*P*spac-pbp1* Δ*pbp1 lacI* Δ*tarO::ery*; Tet^R^, Cm^R^, Ery^R^ | This study |
| *pbp1*_ΔPASTA_ *tarO* | SH1000 *geh::*P*spac-pbp1 pbp1*_ΔPASTA_ *lacI* Δ*tarO::ery*; Tet^R^, Cm^R^, Ery^R^ | This study |
| *pbp1* tarO* | SH1000 *geh::*P*spac-pbp1 pbp1* lacI* Δ*tarO::ery*; Tet^R^, Cm^R^, Ery^R^ | This study |
| *Escherichia coli* | | |
| NEB5α | *fhuA2 (argF-lacZ)U169 phoA glnV44 80 (lacZ)*M15 *gyrA96 recA1 relA1 endA1 thi-1 hsdR17* | New England Biolabs |
| BTH101 | F^−^, cya-99, araD139, galE15, galK16, rpsL1, hsdR2, mcrA1, mcrB1 | (9) |
| Rosetta (DE3) | *F^-^* *ompT hsdS*_B_(r_B_^–^ m_B_^–^) *gal dcm* (DE3) pRARE (Cm^R^) | Novagen |
| BL21(DE3) | F^–^ *omp*T *hsd*S_B_ (r_B_^–^, m_B_^–^) *gal dcm*(DE3) | (10) |

**Table S1B. Plasmids used in this study**

| **Name** | **Characteristics** | **Source** |
| --- | --- | --- |
| pCQ11-FtsZ-SNAP | pCQ11 derivative containing *ftsZ-snap* under P*spac*; Amp^R^, Ery^R^ | (7) |
| pKASBAR | pUC18 containing *attP* and tetracycline cassette; Amp^R^, Tet^R^ | (11) |
| pKB-P*spac*-*pbp1* | pKASBAR containing *S. aureus pbp1* under P*spac*; Amp^R^, Tet^R^ | This study |
| pMAD | *E. coli*-*S. aureus* shuttle vector with temperature-sensitive origin of replication in *S. aureus* and constitutively produced thermostable β-galactosidase encoded by *bgaB*; Amp^R^, Ery^R^ | (12) |
| pMAD-Δ*pbp1* | pMAD containing a deletion cassette for *S. aureus pbp1*; Amp^R^, Ery^R^ | This study |
| pMAD-*pbp1*_ΔPASTA_ | pMAD containing a deletion cassette for *S. aureus pbp1* PASTA domains; Amp^R^, Ery^R^ | This study |
| pMAD-*pbp1** | pMAD containing a cassette for introduction of a point mutation (S314A) in the active site *S. aureus pbp1*; Amp^R^, Ery^R^ | This study |
| pGL485 | *E. coli-S. aureus* shuttle vector carrying *E. coli lacI* gene under the control of a constitutive promoter; Spec^R^, Cam^R^ | (13) |
| T18 (pUT18C) | Derivative of high copy-number pUC19, carrying gene encoding amino acids 225 to 399 of CyaA (T18 fragment); Amp^R^ | (9) |
| T18-zip (pUT18C-zip) | pUT18C coding for the leucine zipper region of the GCN4 yeast protein. Positive control; Amp^R^ | (9) |
| EzrA-T18 (pVF32) | pUT18(9) containing T18 fused in frame to the 3′ end of *S. aureus ezrA*; Amp^R^ | (2) |
| T18-PBP2 (pGL547) | pUT18C containing T18 fused in frame to the 5′ end of *S. aureus pbp2*; Amp^R^ | (2) |
| T18-DivIC (pGL564) | pUT18C containing T18 fused in frame to the 5′ end of *S. aureus divIC*; Amp^R^ | (2) |
| T18-DivIB (pGL544) | pUT18C containing T18 fused in frame to the 5′ end of *S. aureus divIB*; Amp^R^ | (2) |
| T18-FtsW (pALB6) | pUT18C containing T18 fused in frame to the 5′ end of *S. aureus ftsW*; Amp^R^ | (2) |
| T25 (pKT25) | Derivative of low copy-number pSU40, carrying the first 224 amino acids of *B. subtilis* CyaA (T25 fragment); Kan^R^ | (9) |
| T25-zip (pKT25-zip) | pKT25 coding for the leucine zipper region of the GCN4 yeast protein. Positive control; Kan^R^ | (9) |
| T25-PBP1 (pGL550) | pKT25 containing T25 fused in frame to the 5′ end of *S. aureus* *pbp1*; Kan^R^ | (2) |
| T25-PBP1_ΔPASTA_ | pKT25 containing T25 fused in frame to the 5′ end of *S. aureus* *pbp1*_ΔPASTA_ (M1-S595); Kan^R^ | This study |
| pOPINRSF | *kan* P*_T7_ lacI*; Kan^R^ | (14) |
| pVR01 | *kan* P*_T7_ pbp1 lacI*; pOPINRSF derivative for overexpression of full length *S. aureus* PBP1 (1-744); Kan^R^ | This study |
| pVR02 | *kan* P*_T7_ pbp1*(*37-744*) *lacI*; pOPINRSF derivative for overexpression of *Sa*PBP1 (37-744); Kan^R^ | This study. |
| pVR03 | *kan* P*_T7_ pbp1**(*37-744*: S314A) *lacI*; pOPINRSF derivative for overexpression of *Sa*PBP1* (37-744); Kan^R^ | This study |
| pVR04 | *kan* P*_T7_ pbp1*(*37-595*) *lacI*; pOPINRSF derivative for overexpression of *Sa*PBP1_ΔPASTA_(37-595); Kan^R^ | This study |
| pVR06 | *kan* P*_T7_ pbp1*(*595-744*) *lacI*; pOPINRSF derivative for overexpression of *Sa*PASTA_PBP1_ (595-744); Kan^R^ | This study |
| pOPINJB | *bla* P*_T7_ lacI*; Amp^R^ | (14) |
| pSA50 | pOPINJB derivative for overexpression of sPBP1A-BAP; Amp^R^ | This study |

**Table S1C. Oligonucleotides used in this study**

| **Name** | **5**′**-3**′ **oligonucleotide sequence** |
| --- | --- |
| pCQ-pbp1-F | AGAAGGAGATATACATATGGCTTGAGAACGATAATGTAAAG |
| pCQ-pbp1-F | TATTATGCATTTAGAATAGGTTAGTCCGACTTATCCTTG |
| pKB-Pspac-pbp1-F | CCTTTTTTTGCCCCGGGATCCGCAAAAAGTTGTTGACTTTATC |
| pKB-Pspac-pbp1-R | CTATGACCATGATTACGAATTCTTAGTCCGACTTATCCTTG |
| pbp1-A | CCATGGTACCCGGGAGCTCGCACCATGACGCAACATTAG |
| pbp1-B | ATCCTTGTCATTAATTTTTTGCTTCGCC |
| pbp1-C | CAAAAAATTAATGACAAGGATAAGTCGGAC |
| pbp1-D | CCTCGCGTCGGGCGATATCGATCTCCCATAAACACTTTAGC |
| pbp1-E | CCATGGTACCCGGGAGCTCGAATTCTAAAAAACCTAGGCATG |
| pbp1-F | TATCCTTGTCAGATGTGTCATCTTTTGATTTAC |
| pbp1-G | TGACACATCTGACAAGGATAAGTCGGACTAAC |
| pbp1-H | GCGTCTGCAGAAGCTTCTAGTTAATGCACTCCAATCCATAAAC |
| pbp1*5′-F | CCATGGTACCCGGGAGCTCGAATTCAGTATACCGAAGCAACAACCAC |
| pbp1*5′-R | TTAAATGTTGCTCCAGGCTCGTATGTGTTTTG |
| pbp1*3′-F | GAGCCTGGAGCAACATTTAAATCAT ATGGGTTA |
| pbp1*3′-R | CCTCGCGTCGGGCGATATCGGATCCTTAGTCCGACTTATCCTTGTC |
| T25-pbp1-F | CTGCAGGGTCGACTCTAGAGATGGCGAAGCAAAAAATTAAAATTAAAAAAAATAAAATAG |
| T25-pbp1pasta-R | ACGTTGTAAAACGACGGCCGTTAAGATGTGTCATCTTTTGATTTACCTACATTTAAATATTTC |
| VR47F | AAGTTCTGTTTCAGGGCCCGGCGAAGCAGAAGATCAAGATTAAGAAAAAC |
| VR47R | ATGGTCTAGAAAGCTTTAATCGCTTTTATCCTTGTCGGTTTTGC |
| VR49F | ATGATTACCGGCCACAGCAAC |
| VR49R | CGGGCCCTGAAACAGAACTTCCAG |
| VR51 | AACACCTATGAGCCGGGCGCCACCTTCAAAAGCTATGGTC |
| VR53 | GAGCAAAGACGATACCAGCTAAGCGGAGTACAGCAAGG |
| VR57F | AGCAACGCGGAGTACAGCAAGGTGCCGGACGTTG |
| OPPF20018F | AAGTTCTGTTTCAGGGCCCGGCGAACGAGAAATACCTGGTTAAGAACGCGC |
| OPPF20018R | AGATGTCGTTCAGGCCATCGCTTTTATCCTTGTCGGTTTTGCTGTCGC |

**Table S1D. Crystallographic data**

|  | ***Sa*PASTA_PBP1_** |
| --- | --- |
| **Data** |  |
| Resolution (Å) | 1.78 |
| Space group | *P*2_1_2_1_2_1_ |
| *a*, *b*, *c* (Å) | 39.8, 81.4, 89.6 |
| *α*, *β*, *γ* (°) | 90, 90, 90 |
| <I/σI>^a^ | 14.2 (2.0) |
| Completeness (%)^a^ | 99.9 (99.8) |
| Redundancy^a^ | 7.7 (6.7) |
| *R*_p.i.m_ (%)^a^ | 3.0 (39.1) |
| *R*_merge_ (%)^a^ | 6.0 (66.0) |
| CC_1/2_^a^ | 99.8 (92.3) |
|  |  |
| **Refinement** |  |
| *R*_work_ (%) | 18.7 % |
| *R*_free_ (%)^b^ | 21.0 % |
| No. of residues | Chain A: 116, Chain B: 117 |
| No. of waters | 123 |
| Average *B*-factor (Å^2^) |  |
| Protein | Chain A: 45.1, Chain B: 48.0 |
| Waters | 47.6 |
| R.m.s.d on ideal values |  |
| Bond lengths (Å) | 0.005 |
| Bond angle (°) | 0.755 |
|  |  |
| **Ramachandran** |  |
| Most favoured (%) | 98.69 |
| Additional allowed (%) | 1.31 |
| Outliers (%) | 0.0 |
| PDB ID | 7O61 |

^a^Values in parentheses are for the highest resolution shell.

^b^For determination of *R*_free_, 5 % of reflections were randomly selected before refinement.

**References**

1. Horsburgh MJ, Aish JL, White IJ, Shaw L, Lithgow JK, Foster SJ. 2002. σB modulates virulence determinant expression and stress resistance: Characterization of a functional *rsbU* strain derived from *Staphylococcus aureus* 8325-4. Journal of Bacteriology 184:5457–5467.

2. Steele VR, Bottomley AL, Garcia-Lara J, Kasturiarachchi J, Foster SJ. 2011. Multiple essential roles for EzrA in cell division of *Staphylococcus aureus*. Mol Microbiol 80:542–555.

3. Kreiswirth BN, Löfdahl S, Betley MJ, O’Reilly M, Schlievert PM, Bergdoll MS, Novick RP. 1983. The toxic shock syndrome exotoxin structural gene is not detectably transmitted by a prophage. Nature 305:709–712.

4. Lee CY, Buranen SL, Ye ZH. 1991. Construction of single-copy integration vectors for *Staphylococcus aureus*. Gene 103:101–105.

5. Panchal VV, Griffiths C, Mosaei H, Bilyk B, Sutton JAF, Carnell OT, Hornby DP, Green J, Hobbs JK, Kelley WL, Zenkin N, Foster SJ. 2020. Evolving MRSA: High-level β-lactam resistance in S*taphylococcus aureus* is associated with RNA Polymerase alterations and fine tuning of gene expression. PLoS Pathog 16:e1008672.

6. Fey PD, Endres JL, Yajjala VK, Widhelm TJ, Boissy RJ, Bose JL, Bayles KW. 2013. A genetic resource for rapid and comprehensive phenotype screening of nonessential *Staphylococcus aureus* genes. mBio 4:e00537-00512.

7. Lund VA, Wacnik K, Turner RD, Cotterell BE, Walther CG, Fenn SJ, Grein F, Wollman AJ, Leake MC, Olivier N, Cadby A, Mesnage S, Jones S, Foster SJ. 2018. Molecular coordination of S*taphylococcus aureus* cell division. eLife 7:e32057.

8. Salamaga B, Kong L, Pasquina-Lemonche L, Lafage L, Muhlen M von und zur, Gibson JF, Grybchuk D, Tooke AK, Panchal V, Culp EJ, Tatham E, O’Kane ME, Catley TE, Renshaw SA, Wright GD, Plevka P, Bullough PA, Han A, Hobbs JK, Foster SJ. 2021. Demonstration of the role of cell wall homeostasis in *Staphylococcus* aureus growth and the action of bactericidal antibiotics. PNAS 118:e2106022118.

9. Karimova G, Ullmann A, Ladant D. 2001. Protein-protein interaction between *Bacillus stearothermophilus* tyrosyl-tRNA synthetase subdomains revealed by a bacterial two-hybrid system. J Mol Microbiol Biotechnol 3:73–82.

10. Studier FW, Moffatt BA. 1986. Use of bacteriophage T7 RNA polymerase to direct selective high-level expression of cloned genes. J Mol Biol 189:113–130.

11. Bottomley AL, Kabli AF, Hurd AF, Turner RD, Garcia-Lara J, Foster SJ. 2014. *Staphylococcus aureus* DivIB is a peptidoglycan-binding protein that is required for a morphological checkpoint in cell division. Mol Microbiol https://doi.org/10.1111/mmi.12813.

12. Arnaud M, Chastanet A, Débarbouillé M. 2004. New vector for efficient allelic replacement in naturally nontransformable, low-GC-content, gram-positive bacteria. Appl Environ Microbiol 70:6887–6891.

13. Cooper EL, García-Lara J, Foster SJ. 2009. YsxC, an essential protein in *Staphylococcus aureus* crucial for ribosome assembly/stability. BMC Microbiol 9:266.

14. Berrow NS, Alderton D, Owens RJ. 2009. The precise engineering of expression vectors using high-throughput In-Fusion PCR cloning. Methods Mol Biol 498:75–90.
